# The Insecticidal Activity of Secondary Metabolites Produced by *Streptomyces* sp. SA61 against *Trialeurodes vaporariorum* (Hemiptera: Aleyrodidae)

**DOI:** 10.3390/microorganisms12102031

**Published:** 2024-10-08

**Authors:** Fei Liu, Ning Wang, Yinan Wang, Zhiguo Yu

**Affiliations:** 1 College of Plant Protection, Shenyang Agricultural University, Shenyang 110866, China; 15804098223@163.com (F.L.); 13998311869@163.com (N.W.); 2 Biological Invasions Center, Shenyang University, Shenyang 110866, China; yinan7765@163.com

**Keywords:** *Trialeurodes vaporariorum*, *Streptomyces*, strekingmycin, polyketides, insecticidal activity

## Abstract

*Trialeurodes vaporariorum* Westwood poses a significant threat to vegetable and ornamental crops in temperate zones, resulting in notable reductions in yield and substantial economic burdens. In order to find compounds with high insecticidal activity against *T. vaporariorum*, five compounds were isolated and identified from the crude extract of *Streptomyces* sp. SA61. These include three new polyketides, named strekingmycins F–H (**1**–**3**); one new diterpenoid, named phenalinolactone CD8 (**4**); and one known compound, strekingmycin A (**5**). Their structures were analyzed using high-resolution electrospray ionization mass spectrometry and one-dimensional and two-dimensional nuclear magnetic resonance spectroscopy data and by comparing them with previously reported data. The insecticidal activities of compounds **1**–**5** against *T. vaporariorum* were evaluated. Among them, compound **5** exhibited the highest insecticidal activity, with an LC_50_ of 6.949 mg/L against *T. vaporariorum* at 72 h using the leaf-dip method. Lower insecticidal activities were found in compounds **1**–**4**, with LC_50_ values of 22.817, 19.150, 16.981 and 41.501 mg/L, respectively. These data indicate that strekingmycin could be a potential candidate for a novel insecticide to control *T. vaporariorum.*

## 1. Introduction

The *Trialeurodes vaporariorum* Westwood, belonging to the family Aleyrodidae within the order Hemiptera, commonly referred to as the greenhouse whitefly, represents a significant agricultural pest influencing a broad kind of vegetable as well as horticultural crops, particularly in the temperate zones globally [1,2]. It can attack approximately 859 host plant species, which belong to 469 genera in 121 families, demonstrating its adaptability polyphagy [3]. *T*. *vaporariorum* poses a threat to crops through its feeding behavior; the secretion of honeydew that serves as a substrate for fungi, which in turn affects photosynthesis; and its ability to transmit viruses, further compounding the damage inflicted on the plants [4,5,6,7]. Currently, the control of this pest primarily relies on chemical agents such as neonicotinoids, organophosphates, and novel nicotine-based insecticides [8,9,10]. However, the widespread use of synthetic insecticides for managing *T. vaporariorum* populations has led to significant resistance among these pests to a broad range of traditional insecticidal classes, resulting in a decline in the effectiveness of these conventional insecticides [11,12,13]. This phenomenon underscores the necessity for alternative, sustainable approaches to whitefly control that can circumvent the challenges posed by resistance development. Thus, there is a constant demand for novel insecticides to combat the damaging effects of *T*. *vaporariorum*.

Natural compounds boast an extensive heritage serving as a rich repository for bioactive constituents and as a catalyst for the development of numerous pharmaceuticals and agrochemicals [14]. Their extensive spectrum of biological actions, coupled with a plethora of structural variations, underpins their significance in these domains [15,16]. Actinomycetes are renowned for their capacity to generate an extensive array of biologically active secondary metabolites, which have garnered significant applications across various sectors, including industrial processes, medical fields, and agricultural practices. Their ability to produce these diverse compounds positions them as invaluable resources for the development of novel pharmaceuticals, agricultural antibiotics, and other bioactive agents that can address a multitude of challenges in these domains [17].

In our study, the fermentation of *Streptomyces* sp. SA61 and purification of the products led to the isolation of three new polyketides, strekingmycins F–H (**1**–**3**), and one new diterpene, phenalinolactone CD8 (**4**). The results of the insecticidal bioassay revealed that all the tested compounds demonstrated moderate-to-strong lethal activity against *T*. *vaporariorum* (assessed using the leaf-dip method). These findings provide new resources for the development of new microbial insecticides.

## 2. Materials and Methods

### 2.1. General Experimental Procedures

The measurement of optical rotation was conducted under the ATAGO AP-300 polarimeter, manufactured by Atago in Tokyo, Japan. For nuclear magnetic resonance (NMR) spectral acquisition, an Avance-600 Bruker NMR spectrometer, which is produced by Bruker in Karlsruhe, Germany, was utilized under ambient temperature conditions. The chemical shifts were referenced against the carbon and remaining proton signals of chloroform-d (CDCl_3_, with *δ*_C_ 77.0 and *δ*_H_ 7.26) and methanol-d4 (CD_3_OD, with *δ_C_* 49.0 and *δ*_H_ 3.30). HRESIMS data were obtained by using a Waters Xevo G2-XS quadrupole-time-of-flight (Q-TOF) mass spectrometer (Waters, Milford, MA, USA) for spectral analysis. Column chromatography was performed using 100–200-mesh and 200–300-mesh silica gel produced by Qingdao Ocean Spectrum Separation Material Co., Ltd., in Qingdao, China. Additionally, Sephadex LH-20 was employed, which was obtained from GE Healthcare, located in Uppsala, Sweden. Analyses via high-performance liquid chromatography (HPLC) were executed on an Agilent 1260 series instrument, equipped with a ZORBAX Eclipse XDB-C18 column (dimensions: 250 × 4.6 mm, particle size: 5 μm, by Agilent, Santa Clara, CA, USA). For semipreparative scale HPLC separations, an identical Agilent 1260 series system was employed but with a ZORBAX Eclipse XDB-C18 column of larger dimensions (250 × 9.4 mm, particle size: 5 μm, by Agilent). The chemical reagents utilized throughout this research were procured from a supplier, Tianjin Fuyu Fine Chemical Co., Ltd., located in Tianjin, China.

### 2.2. Actinomycete Material

The SA61 strain was isolated from the intestinal tract of earthworms collected at Shenyang Agricultural University, which is located in Shenyang City, Liaoning Province, China [18]. The SA61 strain (GenBank accession no. OR126165) was identified as belonging to the genus *Streptomyces* through morphological analysis (Figure S1) and 16S rRNA sequence analysis (Figure S2) and was stored at China General Microbiological Culture Collection Center (CGMCC) under the accession number of CGMCC No.20306.

### 2.3. Insect Cultures

The initial stock of adult *T. vaporariorum* utilized in this study was obtained from the Laboratory of Insect–Microbe Symbiosis at Shenyang Agricultural University. *T. vaporariorum* was raised on tobacco (*Nicotiana tabacum* CV. Jiyan-9) (Nicotiana: Solanaceae) within a controlled greenhouse environment. The conditions were maintained at a temperature of 27 ± 1 °C, 60 ± 5% humidity, and a photoperiod of 14:12 (light–dark (L:D)). Newly emerged adults were randomly selected for testing.

### 2.4. Fermentation and Extraction

For the subsequent fermentation process, the *Streptomyces* sp. SA61 strain was cultivated on Gause’s No. 1 synthetic agar medium (GS) [19] at 28 °C under the condition of darkness lasting for 7 days. A spore suspension of the cultured strain was prepared using sterile water. Quantitative spore suspensions were added to test tubes (10 × 100 mm), each containing 5 mL of medium ISP2 [20], and incubated at 28 °C while shaking (180 rpm) for 48 h. Subsequently, these mycelial suspensions were moved to 250 mL flasks, each containing 50 mL of medium ISP2, and agitated for 48 h to initiate the seed culture preparation. Ultimately, the seed cultures were transferred into flasks (2 L), each containing 400 mL of medium F [18] supplemented with 16 g of Amberlite XAD-16 resin. After shaking the culture at 28 °C and 180 rpm for 7 d, the fermentation broth was collected to a volume of 48 L. Then, the fermentation broth was washed repeatedly with water, and the resin was collected. The resin was initially subjected to oven-drying at a temperature of 30 °C to eliminate residual moisture, followed by a quadruple extraction process using methanol (MeOH). Subsequently, the MeOH extract was distilled under reduced pressure and dissolved in the a in a 1:1 ratio (0.6 L) in a solution of MeOH and H_2_O. The solution underwent four successive extractions using an equal volume of dichloromethane (CH_2_Cl_2_). The pooled CH_2_Cl_2_ extracts were subsequently concentrated through the application of a rotary evaporation apparatus, yielding 15 g of the crude extract.

### 2.5. Isolation and Purification

The crude extract (15g) was fractionated by normal-phase silica gel column (dimensions: 350 × 25 mm i.d.), employing a stepwise elution strategy utilizing CH_2_Cl_2_–MeOH mixtures (ratios ranging from 100:0 to 1:1, with each gradient constituting 2 L) as the eluent. Four fractions (A–D) were obtained by pooling similar compounds based on thin-layer chromatography (TLC) analysis. Fraction A underwent further purification via a Sephadex LH-20 column, employing a mixture of petroleum ether, dichloromethane, and methanol (in a ratio of 2:1:1) as the eluent, to eliminate impurities. Then, reversed-phase semipreparative HPLC was employed, eluting with a solution containing 84% MeOH–H_2_O, which brought about the isolation of compound **5** (400.5 mg, *t*_R_ = 35.02 min). Fraction C was further fractionated by normal-phase silica gel column chromatography (using 200–300-mesh silica gel), employing a stepwise elution with petroleum ether and ethyl acetate in proportions of 9:1, 8:2, 7:3, 6:4, and 5:5, with each gradient amounting to 1 L. Similar compounds were combined based on TLC analysis, and nine fractions (C1–C9) were collected. Fraction C6 was purified by reversed-phase semipreparative HPLC using 82% MeOH-H_2_O as eluent to yield compound **1** (12.2 mg, *t*_R_ = 26.56 min). In addition, Fraction C7 was purified using 75% MeOH–H_2_O as the eluent, yielding compound **4** (8.2 mg, *t*_R_ = 17.32 min). Fraction B was further fractionated by normal-phase silica gel column (using 200–300-mesh silica gel), with petroleum ether and ethyl acetate ratios of 100:0.5, 100:1, 100:2, 100:4, and 100:8, respectively, yielding a total of seven subfractions (B1–B7), which were obtained by combining similar compounds based on TLC analysis. Fraction B7 was further purified through reversed-phase semipreparative HPLC (77% aqueous CH₃CN), leading to the isolation of compounds **2** (5.5 mg, *t*_R_ = 16.50 min) and **3** (9.6 mg, *t*_R_ = 30.20 min).

Strekingmycin G, **1**: white amorphous powder; ${\left[\alpha \right]}_{D}^{24}-25.13$ (*c* 1.26, CH_3_Cl_3_); HRESIMS *m*/*z* 323.1481 [M + H]^+^ (calcd for C_19_H_31_O_4_, 323.2217);

Strekingmycin F, **2**: white amorphous powder; ${\left[\alpha \right]}_{D}^{24}-72.00$ (*c* 0.40, CH_3_Cl_3_); HRESIMS *m*/*z* 295.2282 [M + H]^+^ (calcd for C_18_H_31_O_3_, 295.2268);

Strekingmycin H, **3**: white amorphous powder; ${\left[\alpha \right]}_{D}^{24}-44.44$ (*c* 0.33, CH_3_Cl_3_); HRESIMS *m*/*z* 351.2199 [M + Na]^+^ (calcd for C_20_H_32_O_4_Na, 351.2198)’

Phenalinolactone CD8, **4**: white amorphous powder; ${\left[\alpha \right]}_{D}^{24}-45.27$ (*c* 0.3, CH_3_OH); HRESIMS *m*/*z* 369.2407 [M + Na]^+^ (calcd for C_22_H_34_O_3_Na, 369.2406).

Strekingmycins G–H (**1**–**3**): for ^1^H NMR and ^13^C NMR data (CDCl_3_), see Table 1 and Table 2; phenalinolactone CD8: for ^1^H NMR and ^13^C NMR (CH_3_OD), see Table 3.

### 2.6. Effect of Compounds on T. vaporariorum Mortality Rates

According to previously reported methods [21], with some modifications, the efficacy of compounds **1**–**5** against *T. vaporariorum* was tested using the leaf-dip method. The tested compounds were dissolved in acetone and diluted with a 0.5% Tween-80 solution to meet the required concentrations of 6.25, 12.5, 25, 50, and 100 mg/L. The trimmed tobacco leaves were immersed in these solutions for 10 s. In contrast, the control leaves were treated with a 0.5% Tween-80 solution, while the positive control leaves were treated with a 2.5 mg/L concentration of acetamiprid.

A total of 30 *T. vaporariorum* adults were introduced into Petri dishes that contained the aforementioned treated leaves. These dishes were then sealed with a plastic film, ensuring that ventilation was maintained through the presence of small perforations. Each treatment was conducted with three replicates. The mortality of *T*. *vaporariorum* was examined at 24, 48, and 72 h after treatment. The corrected mortality rate was calculated using the following formula [22]:

corrected mortality (%) = (P1 − T0)/(100 − P0) × 100

where P1 is the mortality rate in the treatment group; P0 is the mortality rate in the control group.

## 3. Results

### 3.1. Structure Elucidation of Compounds

**Figure 1:**
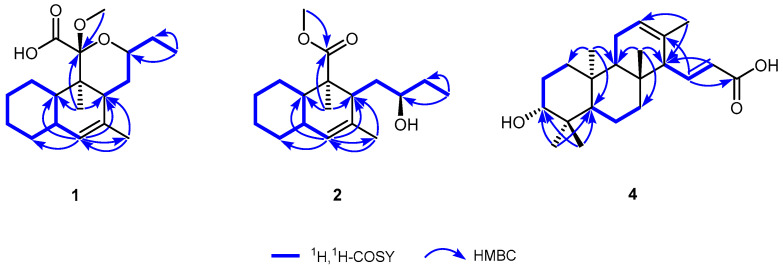
^1^H-^1^H COSY and significant HMBCs of compounds **1**, **2,** and **4**. ^1^H-^1^H COSY—H-H correlation spectroscopy; HMBC—heteronuclear single-quantum correlation.

**Figure 2:**
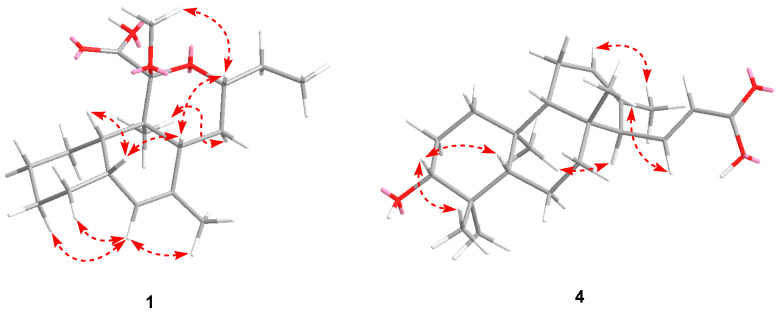
Key NOESY correlations of compounds **1** and **4**. NOESY—nuclear Overhauser effect spectroscopy.

**Figure 3:**
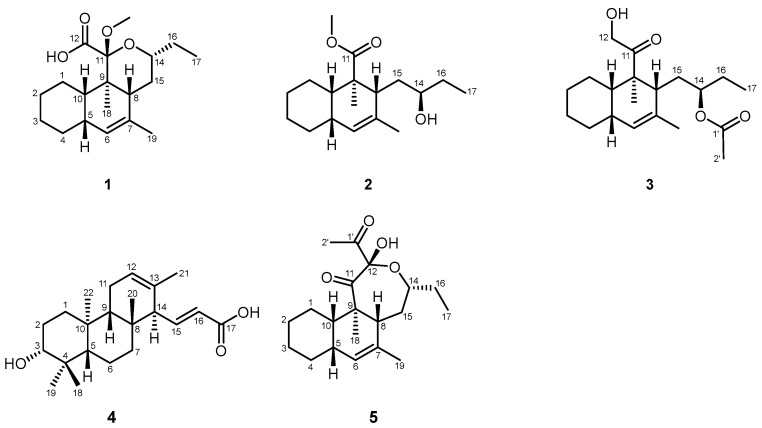
Chemical structures of compounds **1**–**5**.

Compound **1** was obtained as a white amorphous powder. Its molecular formula is C_19_H_30_O_4_, as determined by positive HRESIMS, indicating five indices of hydrogen deficiency. The ^1^H NMR spectrum of **1** in CDCl_3_, as detailed in Table 1, exhibited a broad, exchangeable singlet peak at *δ*_H_ 9.25 (1H, br s, COOH). Additionally, a signal characteristic of an olefinic proton was observed at *δ*_H_ 5.55 (1H, r s, H-6). The spectral data additionally revealed a signal attributable to an oxygen-bearing methine proton at *δ*_H_ 3.70 (1H, m, H-14), a clear signal corresponding to a methoxy group at *δ*_H_ 3.27 (3H, s), and a signal indicative of an olefinic methyl group at *δ*_H_ 1.68 (3H, br s, H-19). Additionally, two aliphatic methyl groups were discerned by their signals in the high-field area: one manifesting as a singlet and the other as a triplet. The ^13^C NMR spectroscopic data, presented in Table 2, encompassed 19 distinct carbon resonances. Among these, a singlet at *δ*_C_ 169.1 was attributed to the carbonyl carbon of a carboxyl group (C-12). The presence of two olefinic carbons was denoted by signals at *δ*_C_ 136.2 (s, C-7) and 128.3 (d, C-6). A ketal carbon was identified by its singlet at *δ*_C_ 104.4 (C-11), while an oxygen-bearing methine carbon was indicated by a doublet at *δ*_C_ 74.0 (C-14). A methoxy carbon was represented by a quartet at *δ*_C_ 51.3. The remaining signals corresponded to 13 aliphatic carbons in the high-field region. The occurrence of a double bond and a carbonyl moiety explain the two indices of hydrogen deficiency, which is suggestive of a tricyclic structural framework in **1**. The NMR spectroscopic characteristics previously delineated indicate that **1** is likely a tricyclic polyketide, structurally akin to *streptomyces*-derived strekingmycin D, and is biosynthesized by the identical strain of *Streptomyces* [18]. This inference is drawn from the observed NMR data, which align with the known structural features of strekingmycin D, a compound known to be produced by a specific *Streptomyces* species. The similarity in the NMR profiles further supports the hypothesis of a common biosynthetic pathway and structural homology between the two compounds. An analysis of the NMR spectra of **1** in relation to strekingmycin D demonstrated that the apparent difference only originated from the ring C moiety: the acetyl group of the latter was absent and replaced by a carboxyl group. The heteronuclear multiple-bond correlation (HMBC) spectrum, as depicted in Figure 1, revealed a significant correlation between the methoxy proton signal and the ketal carbon, thereby affirming the existence of the methoxy group at the C-11 position. The two-dimensional NMR analysis provided further validation for the planar structure, with particular emphasis on ring C moiety. Furthermore, the nuclear Overhauser effect spectroscopy (NOESY) data, illustrated in Figure 2, indicated correlations such as H-5↔H-8/H-10, H-14↔H-8/OCH3, and H3-18↔H-15α. These NOESY correlations were instrumental in establishing the relative stereochemistry of **1**, which was found to be in accordance with that of strekingmycin D. The absolute configuration was also deduced to be the same as that of strekingmycin D in view of the same biological source. Thus, the structure of **1** was established as shown in Figure 3 and named strekingmycin G.

Compound **2** was obtained as a white amorphous powder. Its molecular formula is C_18_H_30_O_3_, as determined by positive HRESIMS, indicating four indices of hydrogen deficiency. The ^1^H NMR data for **2** in CDCl_3_, as listed in Table 1, exhibited the existence of an olefinic proton resonating at *δ*_H_ 5.01 (1H, br s, H-6). Additionally, a signal corresponding to a methoxy group was detected at *δ*_H_ 3.65 (3H, s), as well as an oxygen-bearing methine proton signal at *δ*_H_ 3.52 (1H, m, H-14). Furthermore, it featured a broad singlet attributed to an olefinic methyl group, along with two distinguishable aliphatic methyl signals, with one appearing as a singlet and the other as a triplet. The ^13^C NMR spectrum extremely resembles that of **1**, as detailed in Table 2, just missing the ketal carbon signal. Taking into account the NMR spectroscopic characteristics and the index of hydrogen deficiency, it is plausible to infer that **2** represents a bicyclic polyketide, potentially resulting from the cleavage of ring C in **1**. The HMBCs of the methoxy proton and H_3_-18 [*δ*_H_ 1.26 (3H, s)] to a carbonyl carbon at *δ*_C_ 179.1 (s, C-11), as shown in Figure 1, revealed a methyl carboxylate fragment at C-9. The secondary alcohol, presumed to originate from the ring cleavage process, was anticipated to retain its original absolute configuration without alteration. Consequently, the definitive structure of **2** was delineated as illustrated in Figure 3 and named strekingmycin F.

Compound **3** was obtained as a white amorphous powder. Its molecular formula is C_20_H_32_O_4_, as determined by positive HRESIMS. The ^1^H NMR data for **3** in CDCl_3_, listed in Table 1, displayed an olefinic proton resonating at *δ*_H_ 5.02 (1H, br s, H-6), an oxygen-bearing methine proton resonating at *δ*_H_ 4.85 (1H, m, H-14), an oxygenated methylene signal at *δ*_H_ 4.28, 4.37 (each 1H, d, *J* = 18.3 Hz, H-12), an exchangeable broad singlet at *δ*_H_ 3.24 (1H, br s, 12-OH), a signal indicative of an acetoxy group at *δ*_H_ 2.04 (3H, s), an olefinic methyl broad singlet, and two aliphatic methyl signals (one singlet and one triplet). The ^13^C NMR spectrum, as documented in Table 2, indicated a total of 20 carbon resonances, containing 1 ketone carbonyl carbon, 1 acetoxy carbonyl carbon, 2 olefinic carbons, and 2 oxygenated carbons (1 methine and 1 methylene). Upon conducting a comparative NMR data analysis between **3** and **2**, it was found that the methyl carboxylate group present in **2** was notably absent in **3**, and an acetoxy group was newly detected. The HMBC indicated a notable correlation between the oxygenated methylene protons and H_3_-18 to the ketone carbon resonance at *δ*_C_ 215.2 (s, C-11). This observation confirmed the presence of a 2-hydroxyacetyl at the C-9 position, while the hydroxy at C-14 was acetylated, as evidenced by a dramatic downfield shift of H-14. The stereochemistry of **3** was confirmed to be consistent with that of **2** through analysis of the NOESY correlations. Ultimately, as depicted in Figure 3, the definitive structure of **3** was elucidated and consequently designated as strekingmycin H.

Compound **4** was obtained as a white amorphous powder. Its molecular formula is C_22_H_34_O_3_, as identified by positive HRESIMS, indicating six indices of hydrogen deficiency. The ^1^H NMR data for **4** in CD_3_OD, as listed in Table 3, revealed a pair of olefinic protons that exhibited mutual coupling, appearing at *δ*_H_ 6.68 (1H, dd, *J* = 15.6, 10.3 Hz, H-15) and 5.89 (1H, d, *J* = 15.6 Hz, H-16), indicative of a trans-configured double bond. Additionally, the spectrum included a signal attributed to an oxygenated methine proton at *δ*_H_ 3.12 (1H, dd, *J* = 11.6, 4.6 Hz, H-3), an olefinic methyl signal at *δ*_H_ 1.53 (3H, br s, H-21), and four methyl singlets in the high-field region. The ^13^C NMR spectrum (Table 3) showed a total of twenty-two carbon signals, including one carboxyl carbonyl carbon at *δ*_C_ 171.0 (s, C-17), four olefinic carbons due to two double bonds, and one oxygenated methine carbon at *δ*_C_ 79.6 (d, C-3), as well as three quaternary carbons, three methine carbons, five methylene carbons, and five methyl carbons in the high-field region. The features of the previously mentioned NMR were extremely comparable to those of phenalinolactone CD6, a rare diterpenoid antibiotic produced by a *Streptomyces* strain [23], and a distinguishing feature was noted in the absence of the carbonyl carbon signal characteristic of a ketone group in the structure of phenalinolactone CD6. The HMBCs (Figure 1) from H-15 at *δ*_H_ 6.68 (1H, dd, *J* = 15.6, 10.3 Hz) to the carboxyl carbon at *δ*_C_ 171.0 (s, C-17) and C-13 [*δ*_C_ 133.3 (s)] led us to the conclusion that the substituent at C-14 was an (E)-acrylic acid moiety. The resulting planar structure was further confirmed by the 2D NMR analysis. The relative configuration of **4** was determined to be the same as that of phenalinolactone CD6 according to the NOESY correlations (Figure 2) of H-3↔H-5/H3-18, H-14↔H3-22, and H-15↔H3-20. Thus, the structure of **4** was established as shown in Figure 3 and named phenalinolactone CD8. The biosynthetic pathway for the new metabolite may involve extension at C-15 of the diterpene part with acetyl-CoA and then C-15–C-16 dehydrogenation.

### 3.2. Insecticidal Activity Assay

The insecticidal effects of compounds **1**–**5** on *T*. *vaporariorum* were assessed using the leaf-dip methods. The findings revealed the insecticidal efficacies of the quintet of compounds when tested on *T. vaporariorum* at 24, 48, and 72 h were substantially different when measured using the leaf-dip method (Table 4 and Figure S39). Among them, compound **5** exhibited the highest activity against *T*. *vaporariorum*, with a corrected mortality rate of 89.74 ± 0.82% after 72 h of treatment, comparable to acetamiprid. The corrected mortality rates of the other four compounds against *T*. *vaporariorum* at 72 h ranged from 80.66 ± 2.43% to 56.62 ± 5.3%, which were lower than the positive control.

## 4. Discussion

Five compounds were separated from the crude extract of *Streptomyces* sp. SA61. Among them, compounds **1**, **2**, **3**, and **5** are classified as polyketides. Polyketides are constructed through repetitive decarboxylative condensation reactions of basic two-carbon acetate units, similar to the biosynthesis of fatty acids. This includes the use of the same simple precursor pool, such as acetyl coenzyme A (CoA) and malonyl coenzyme A (MCoA) units. [24,25]. Polyketides represent a large class of natural products with various physiological functions and are particularly valuable in pharmacology, such as antibiotics and immunosuppressants [25,26]. In the field of biopesticides, polyketides synthesized by actinomycetes represent a crucial class of biologically active compounds known for their insecticidal properties. These compounds are also prime candidates for applications in agriculture and animal husbandry [27]. As a crucial subset of actinomycetes, the isolation and characterization of *Streptomyces* are essential steps in the discovery of biologically active secondary metabolites [28]. In our study, the fermented extracts and new isolates of *Streptomyces* sp. SA61 showed potent activity against *T. vaporariorum*, indicating that these products can be used as natural pest control agents.

Natural products have garnered widespread attention due to their environmental safety and low toxicity to organisms. *Streptomyces* is considered a reservoir of various natural products, owing to its powerful and complex metabolic capabilities. Many of its secondary metabolites, which exhibit insecticidal activity, have been widely studied. For example, doramectin congeners produced by *Streptomyces avermitilis* NEAU1069 have an IC_50_ value of 10.2 mg/mL against adult two-spotted spider mites (*Tetranychus urticae Koch*) [29]. Similarly, endostemonines A-J, produced by the endophytic *Streptomyces* sp. BS-1, all exhibited potent lethal activity against *Aphis gossypii*, with LC_50_ values ranging from 3.55 to 32.00 mg/L after 72 h [30]. Additionally, tartrolone C, produced by *Streptomyces* sp. CP1130, exhibited inhibitory effects on both beet armyworm and tobacco budworm, with a MELC^10^ value of 125 ppm for both insects [31]. Furthermore, the new nemadectin congener Seco-nemadectin, produced by *Streptomyces microflavus* neau3, exhibited nematicidal activity against *Caenorhabditis elegans*, with an LC_50_ value of 15.4 µg/mL after 15 h of treatment [32]. In the current study, a series of compounds, including strekingmycin A, strekingmycins F–H, and phenalinolactone CD8, were successfully extracted from the metabolites produced by the *Streptomyces* sp. SA61 during its fermentation process. These compounds exhibited significant insecticidal activity against *Trialeurodes vaporariorum* in the leaf-dip method. The LC_50_ values for this method ranged from 6.949 to 41.501 mg/L. Given their structural uniqueness and high activity, these compounds have the potential to serve as lead compounds for insecticide development.

Compounds **1**, **2**, **3**, and **5** exhibited high sensitivity against *T. vaporariorum*. Previous research demonstrated that compound **5** also exhibited significant insecticidal activity against *Myzus persicae* (Sulzer), with an LC_50_ value of 7.09 mg/L. This suggests that these compounds contain a wide-ranging spectrum of insecticidal activity. Moreover, these compounds feature an extremely unique skeleton, and no similarly structured insecticides have been reported [33]. Given the unique structural features and notable bioactivity of strekingmycin, these substances are promising candidates as precursors for the development of insecticidal agents. Additionally, it is imperative to conduct further assessments to determine the effectiveness and operational mechanisms of strekingmycin against a variety of pest species.

## 5. Conclusions

To conclude, the crude extract derived from the fermentation of *Streptomyces* sp. SA61 demonstrated significant efficacy in targeting *T. vaporariorum*. Further, compounds **1**–**4** were isolated and identified from *Streptomyces* sp. SA61 for the first time, with **1**–**3** considered novel polyketides and **4** identified as a new diterpenoid. Additionally, compounds **2**, **3**, and **5** showed considerable efficacy against *T. vaporariorum*. Given the remarkable insecticidal activity often found in microbial secondary metabolites, they have the potential to be considered as precursors for the formulation of insecticidal agents in agriculture.

## Figures and Tables

**Table 1:** ^1^H NMR spectral data for compounds **1**–**3** in CDCl_3_^a^.

No.	1	2	3
1	0.76 (1H_α_, qd-like, 12.6, 3.3) 1.38 (1H_β_, br d, 12.6)	1.14 (1H_α_, m) 1.55 (1H_β_, m)	1.14 (1H_α_, m) 1.58 (1H_β_, m)
2	1.25 (1H_β_, m) 1.60 (1H_α_, m)	1.14 (1H_β_, m) 1.65 (1H_α_, m)	1.15 (1H_β_, m) 1.69 (1H_α_, m)
3	1.20 (1H_α_, m) 1.49 (1H_β_, br d, 12.3)	1.21 (1H_α_, m) 1.39 (1H_β_, m)	1.17 (1H_α_, m) 1.43 (1H_β_, m)
4	1.62 (1H_β_, m) 1.79 (1H_α_, br d, 13.3)	1.49 (1H_β_, m) 1.57 (1H_α_, m)	1.47 (1H_β_, m) 1.58 (1H_α_, m)
5	2.27 (1H, br s)	2.18 (1H, br s)	2.09 (1H, br s)
6	5.55 (1H, br s)	5.01 (1H, br s)	5.02 (1H, br s)
8	2.77 (1H, br d, 13.0)	2.63 (1H, br s)	2.29 (1H, br s)
10	2.62 (1H, dt-like, 13.0, 5.1)	1.99 (1H, m)	1.95 (1H, m)
12	—	—	4.28, 4.37 (each 1H, d, 18.3)
14	3.70 (1H, m)	3.52 (1H, m)	4.85 (1H, m)
15	1.63 (1H_β_, m) 1.73 (1H_α_, q-like, 12.8)	1.43, 1.61 (each 1H, m)	1.48 (1H, ddd, 15.3, 5.3, 4.0) 1.86 (1H, ddd, 15.3, 9.5, 2.9)
16	1.60–1.69 (2H, m)	1.42, 1.56 (each 1H, m)	1.53–1.64 (2H, m)
17	1.02 (3H, t, 7.4)	0.96 (3H, t, 7.4)	0.91 (3H, t, 7.4)
18	0.72 (3H, s)	1.26 (3H, s)	1.19 (3H, s)
19	1.68 (3H, br s)	1.74 (3H, br s)	1.79 (3H, br s)
OCH_3_	3.27 (3H, s)	3.65 (3H, s)	—
CH_3_CO	—	—	2.04 (3H, s)

^a^ NMR data (*δ*) were recorded at 600 MHz for ^1^H and at 150 MHz for ^13^C in CDCl_3_. NMR—nuclear magnetic resonance.

**Table 2:** ^13^C NMR spectral data for compounds **1**–**3** in CDCl_3_.

No.	1	2	3
1	27.9 (t)	23.7 (t)	23.6 (t)
2	26.7 (t)	26.3 (t)	26.4 (t)
3	22.6 (t)	22.7 (t)	22.5 (t)
4	30.3 (t)	31.5 (t)	31.4 (t)
5	35.4 (d)	35.0 (d)	34.5 (d)
6	128.3 (d)	124.5 (d)	125.2 (d)
7	136.2 (s)	137.5 (s)	137.1 (s)
8	38.3 (d)	40.4 (d)	38.5 (d)
9	45.1 (s)	50.6 (s)	53.8 (s)
10	38.5 (d)	40.9 (d)	41.1 (d)
11	104.4 (s)	179.1 (s)	215.2 (s)
12	169.1 (s)	—	64.2 (t)
14	74.0 (d)	74.9 (d)	77.0 (d)
15	28.3 (t)	38.1 (t)	34.9 (t)
16	28.6 (t)	30.5 (t)	27.5 (t)
17	10.0 (q)	10.2 (q)	9.8 (q)
18	14.0 (q)	20.3 (q)	20.4 (q)
19	19.0 (q)	22.7 (q)	22.6 (q)
OCH_3_	51.3 (q)	51.9 (q)	—
CH_3_CO	—	—	171.0 (s)
CH_3_CO	—	—	21.2 (q)

**Table 3:** ^1^H and ^13^C NMR spectral data for compound **4** in CD_3_OD ^a^.

No.	^1^H NMR	^13^C NMR
1	0.95 (1H_β_, m) 1.81 (1H_α_, br d, 13.0)	40.3 (t)
2	1.57, 1.63 (each 1H, m)	28.0 (t)
3	3.12 (1H, dd, 11.6, 4.6)	79.6 (d)
4	—	39.9 (s)
5	0.85 (1H, m)	57.0 (d)
6	1.46–1.54 (2H, m)	19.0 (t)
7	1.07 (1H_β_, m) 1.81 (1H_α_, br d, 13.0)	39.9 (t)
8	—	37.4 (s)
9	1.05 (1H, m)	56.0 (d)
10	—	39.3 (s)
11	2.09 (1H_α_, br d, 19.0) 2.22 (1H_β_, br d, 19.0)	23.5 (t)
12	5.49 (1H, br s)	123.6 (d)
13	—	133.3 (s)
14	3.05 (1H, br d, 10.3)	48.7 (d)
15	6.68 (1H, dd, 15.6, 10.3)	150.8 (d)
16	5.89 (1H, d, 15.6)	127.5 (d)
17	—	171.0 (s)
18	0.96 (3H, s)	28.8 (q)
19	0.78 (3H, s)	16.3 (q)
20	0.89 (3H, s)	27.8 (q)
21	1.53 (3H, br s)	23.2 (q)
22	0.93 (3H, s)	15.1 (q)

^a^ Overlapped in the solvent signal. NMR data (*δ*) were recorded at 600 MHz for ^1^H and at 150 MHz for ^13^C in CD_3_OD.

**Table 4:** The potency of compounds **1**–**5** on *T. vaporariorum* at 72 h ^a^.

Compound	Toxicity Regression Equation (y)	LC_50_ (95% CL) (mg/L)	χ^2^	*p* Value
Strekingmycin G, **1**	Y = −1.915 + 1.410x	22.817 (19.090–27.144)	9.990	0.695
Strekingmycin H, **2**	Y = −1.982 + 1.546x	19.150 (16.132–22.459)	17.927	0.160
Strekingmycin F, **3**	Y = −2.248 + 1.827x	16.981 (14.587–19.521)	16.235	0.237
Phenalinolactone CD8, **4**	Y = −2.089 + 1.291x	41.501 (34.149–52.257)	7.976	0.845
Strekingmycin A, **5**	Y = −1.521 + 1.805x	6.949 (5.415–8.456)	10.116	0.684

^a^ LC_50_, 50% lethal concentration; CL, confidence limit; χ^2^, chi-squared value. Data are the average of three replicates and have been adjusted for control mortality using the Abbott formula [22].

## Data Availability

The original contributions presented in the study are included in the article/Supplementary Material, further inquiries can be directed to the corresponding author.

## Supplementary Materials

The following supporting information can be downloaded at: https://www.mdpi.com/article/10.3390/microorganisms12102031/s1, Figure S1: Spore morphology of strain SA61 was observed by scanning electron microscopy; Figure S2: Morphological characteristics of strain SA61 on different media; Figure S3: The phylogenetic tree of strain SA61; Figures S4–S38: NMR (^1^H-NMR, ^13^C-NMR, HSQC, HMBC, ^1^H-^1^H COSY, and NOESY) and HRESIMS spectrum of compounds **1**–**5**; Figure S39: The insecticidal activity of compounds **1**–**5** against *T. vaporariorum* was evaluated using the leaf-dip method.

## References

[1] Choi W.I., Lee E.H., Choi B.R., Park H.M., Ahn Y.J. (2003). Toxicity of plant essential oils to Trialeurodes vaporariorum (Homoptera: Aleyrodidae). J. Econ. Entomol..

[2] Mendoza-García E.E., Ortega-Arenas L.D., Pérez-Pacheco R., Rodríguez-Hernández C. (2014). Repellency, toxicity, and oviposition inhibition of vegetable extracts against greenhouse whitefly trialeurodes vaporariorum (westwood) (hemiptera: Aleyrodidae). Chil. J. Agric. Res..

[3] CABI (2021). Invasive Species Compendium. Trialeurodes vaporariorum. (Whitefly, Greenhouse). http://www.cabi.org/isc/datasheet/54660.

[4] Moodley V., Gubba A., Mafongoya P.L. (2019). A survey of whitefly-transmitted viruses on tomato crops in South Africa. Crop Prot..

[5] Jones D.R. (2003). Plant viruses transmitted by whiteflies. Eur. J. Plant Pathol..

[6] Hanssen I.M., Lapidot M. (2012). Major tomato viruses in the Mediterranean basin. Adv. Virus Res..

[7] Perring T.M., Stansly P.A., Liu T.X., Smith H.A., Andreason S.A., Wakil W., Brust G.E., Perring T.M. (2018). Whiteflies: Biology, Ecology, and Management. In Sustainable Management of Arthropod Pests of Tomato.

[8] Gorman K., Devine G., Bennison J., Coussons P., Punchard N., Denholm I. (2007). Report of resistance to the neonicotinoid insecticide imidacloprid in Trialeurodes vaporariorum (Hemiptera: Aleyrodidae). Pest. Manag. Sci..

[9] Millar N.S., Denholm I. (2007). Nicotinic acetylcholine receptors: Targets for commercially important insecticides. Invert. Neurosci..

[10] Soderlund D.M., Bloomquist J.R. (1989). Neurotoxic actions of pyrethroid insecticides. Annu. Rev. Entomol..

[11] Bi J.L., Toscano N.C. (2007). Current status of the greenhouse whitefly, Trialeurodes vaporariorum, susceptibility to neonicotinoid and conventional insecticides on strawberries in southern California. Pest. Manag. Sci..

[12] Kapantaidaki D.E., Sadikoglou E., Tsakireli D., Kampanis V., Stavrakaki M., Schorn C., Ilias A., Riga M., Tsiamis G., Nauen R. (2018). Insecticide resistance in Trialeurodes vaporariorum populations and novel diagnostics for kdr mutations. Pest. Manag. Sci..

[13] Gorman K., Hewitt F., Denholm I., Devine G.J. (2002). New developments in insecticide resistance in the glasshouse whitefly (Trialeurodes vaporariorum) and the two-spotted spider mite (Tetranychus urticae) in the UK. Pest. Manag. Sci..

[14] Katz L., Baltz R.H. (2016). Natural product discovery: Past, present, and future. J. Ind. Microbiol. Biotechnol..

[15] Lorsbach B.A., Sparks T.C., Cicchillo R.M., Garizi N.V., Hahn D.R., Meyer K.G. (2019). Natural products: A strategic lead generation approach in crop protection discovery. Pest. Manag. Sci..

[16] Sparks T.C., Sparks J.M., Duke S.O. (2023). Natural Product-Based Crop Protection Compounds—Origins and Future Prospects. J. Agric. Food Chem..

[17] Barka E.A., Vatsa P., Sanchez L., Gaveau-Vaillant N., Jacquard C., Klenk H.P., Clément C., Ouhdouch Y., van Wezel G.P. (2015). Taxonomy, Physiology, and Natural Products of Actinobacteria. Microbiol. Mol. Biol. Rev..

[18] Wang N., Zhu K., Bi Y., Liu F., Yu Z. (2023). Anti-Aphid Polyketides from *Streptomyces* sp. SA61. J. Nat. Prod..

[19] Waksman S.A. (1961). Classification, identification and descriptions of genera and species. The Actinomycetes.

[20] Shirling E.T., Gottlieb D. (1966). Methods for characterization of Streptomyces specie. Int. J. Syst. Evol. Microbiol..

[21] Cao C.W., Zhang J., Gao X.W., Liang P., Guo H.L. (2008). Overexpression of carboxylesterase gene associated with organophosphorous insecticide resistance in cotton aphids, Aphis gossypii (Glover). Pestic. Biochem. Physiol..

[22] Abbott W.S. (1987). A method of computing the effectiveness of an insecticide. J. Am. Mosq. Control Assoc..

[23] Dürr C., Schnell H.J., Luzhetskyy A., Murillo R., Weber M., Welzel K., Vente A., Bechthold A. (2006). Biosynthesis of the terpene phenalinolactone in Streptomyces sp. Tü6071: Analysis of the gene cluster and generation of derivatives. Chem. Biol..

[24] Shimizu Y., Ogata H., Goto S. (2017). Type III Polyketide Synthases: Functional Classification and Phylogenomics. Chembiochem.

[25] Hertweck C. (2009). The biosynthetic logic of polyketide diversity. Angew. Chem. Int. Ed. Engl..

[26] Chooi Y.H., Tang Y. (2012). Navigating the fungal polyketide chemical space: From genes to molecules. J. Org. Chem..

[27] Toopaang W., Bunnak W., Srisuksam C., Wattananukit W., Tanticharoen M., Yang Y.L., Amnuaykanjanasin A. (2022). Microbial polyketides and their roles in insect virulence: From genomics to biological functions. Nat. Prod. Rep..

[28] Montesinos E. (2003). Development, registration and commercialization of microbial pesticides for plant protection. Int. Microbiol..

[29] Wang X.J., Zhang J., Wang J.D., Huang S.X., Chen Y.H., Liu C.X., Xiang W.S. (2011). Four new doramectin congeners with acaricidal and insecticidal activity from Streptomyces avermitilis NEAU1069. Chem. Biodivers..

[30] Zhao H., Yang A., Zhang N., Li S., Yuan T., Ding N., Zhang S., Bao S., Wang C., Zhnag Y. (2020). Insecticidal Endostemonines A-J Produced by Endophytic Streptomyces from Stemona sessilifolia. J. Agric. Food Chem..

[31] Lewer P., Chapin E.L., Graupner P.R., Gilbert J.R., Peacock C. (2003). Tartrolone C: A novel insecticidal macrodiolide produced by *Streptomyces* sp. CP1130. J. Nat. Prod..

[32] Xiang W.S., Wang J.D., Wang M., Wang X.J. (2010). New nemadectin congener from Streptomyces microflavus neau3: Fermentation, separation, structure elucidation and biological activities. J. Antibiot..

[33] Sparks T.C., Nauen R. (2015). IRAC: Mode of action classification and insecticide resistance management. Pestic. Biochem. Physiol..

