# Supplementary material for: The Insecticidal Activity of Secondary Metabolites Produced by Streptomyces sp. SA61 against Trialeurodes vaporariorum (Hemiptera: Aleyrodidae)
Source: Microorganisms. 2024 Oct 8;12(10):2031. doi: 10.3390/microorganisms12102031 (PMC11509760; doi:10.3390/microorganisms12102031)

# Supporting information

# The Insecticidal Activity of Secondary Metabolites Produced by *Streptomyces* sp. SA61 against *Trialeurodes vaporariorum* (Hemiptera: Aleyrodidae)

**Fei Liu,[1] Ning Wang,[1] Yinan Wang[1] and Zhiguo Yu[1,2,*]**

[1]College of Plant Protection, Shenyang Agricultural University, Shenyang 110866, China; 15804098223@163.com (F.L.); 13998311869@163.com (N.W.); yinan7765@163.com (Y.W.)

[2]Engineering & Technological Research Center of Biopesticide for Liaoning Province, Shenyang 110866, China

[*]Correspondence: zyu@syau.edu.cn; Tel.: +86-24-88487148

**Contents**

**Figure S1.** Spore morphology of strain SA61 was observed by scanning electron microscopy ........ 3
**Figure S2.** Morphological characteristics of strain SA61 on different media ........ 3
**Figure S3.** Neighbour-joining phylogenetic tree based on the 16S rRNA gene sequences with members of the genus *Streptomyces* ........ 4
**Figure S4.** The $^{1}$H-NMR spectrum of compound **1** in $CDCl_3$ ........ 5
**Figure S5.** The $^{13}$C-NMR spectrum of compound **1** in $CDCl_3$ ........ 5
**Figure S6.** The HSQC spectrum of compound **1** in $CDCl_3$ ........ 6
**Figure S7.** The HMBC spectrum of compound **1** in $CDCl_3$ ........ 6
**Figure S8.** The $^{1}$H-$^{1}$H COSY spectrum of compound **1** in $CDCl_3$ ........ 7
**Figure S9.** The NOESY spectrum of compound **1** in $CDCl_3$ ........ 7
**Figure S10.** The HRESIMS spectrum of compound **1** ........ 8
**Figure S11.** The $^{1}$H-NMR spectrum of compound **2** in $CDCl_3$ ........ 8
**Figure S12.** The $^{13}$C-NMR spectrum of compound **2** in $CDCl_3$ ........ 9
**Figure S13.** The HSQC spectrum of compound **2** in $CDCl_3$ ........ 9
**Figure S14.** The HMBC spectrum of compound **2** in $CDCl_3$ ........ 10
**Figure S15.** The $^{1}$H-$^{1}$H COSY spectrum of compound **2** in $CDCl_3$ ........ 10
**Figure S16.** The NOESY spectrum of compound **2** in $CDCl_3$ ........ 11
**Figure S17.** The HRESIMS spectrum of compound **2** ........ 11
**Figure S18.** The $^{1}$H-NMR spectrum of compound **3** in $CDCl_3$ ........ 12
**Figure S19.** The $^{13}$C-NMR spectrum of compound **3** in $CDCl_3$ ........ 12
**Figure S20.** The HSQC spectrum of compound **3** in $CDCl_3$ ........ 13
**Figure S21.** The HMBC spectrum of compound **3** in $CDCl_3$ ........ 13
**Figure S22.** The $^{1}$H-$^{1}$H COSY spectrum of compound **3** in $CDCl_3$ ........ 14
**Figure S23.** The NOESY spectrum of compound **3** in $CDCl_3$ ........ 14
**Figure S24.** The HRESIMS spectrum of compound **3** ........ 15
**Figure S25.** The $^{1}$H-NMR spectrum of compound **4** in $CDCl_3$ ........ 15
**Figure S26.** The $^{13}$C-NMR spectrum of compound **4** in $CDCl_3$ ........ 16
**Figure S27.** The HSQC spectrum of compound **4** in $CDCl_3$ ........ 16
**Figure S28.** The HMBC spectrum of compound **4** in $CDCl_3$ ........ 17
**Figure S29.** The $^{1}$H-$^{1}$H COSY spectrum of compound **4** in $CDCl_3$ ........ 17
**Figure S30.** The NOESY spectrum of compound **4** in $CDCl_3$ ........ 18
**Figure S31.** The HRESIMS spectrum of compound **4** ........ 18
**Figure S32.** The $^{1}$H-NMR spectrum of compound **5** in $CDCl_3$ ........ 19
**Figure S33.** The $^{13}$C-NMR spectrum of compound **5** in $CDCl_3$ ........ 19
**Figure S34.** The HSQC spectrum of compound **5** in $CDCl_3$ ........ 20
**Figure S35.** The HMBC spectrum of compound **5** in $CDCl_3$ ........ 20
**Figure S36.** The $^{1}$H-$^{1}$H COSY spectrum of compound **5** in $CDCl_3$ ........ 21
**Figure S37.** The NOESY spectrum of compound **5** in $CDCl_3$ ........ 21
**Figure S38.** The HRESIMS spectrum of compound **5** ........ 22
**Figure S39.** The insecticidal activity of compounds **1–5** against *T. vaporariorum* was evaluated using the leaf-dip method.. ........ 23

**Figure S1.** Spore morphology of strain SA61 was observed by scanning electron microscopy.

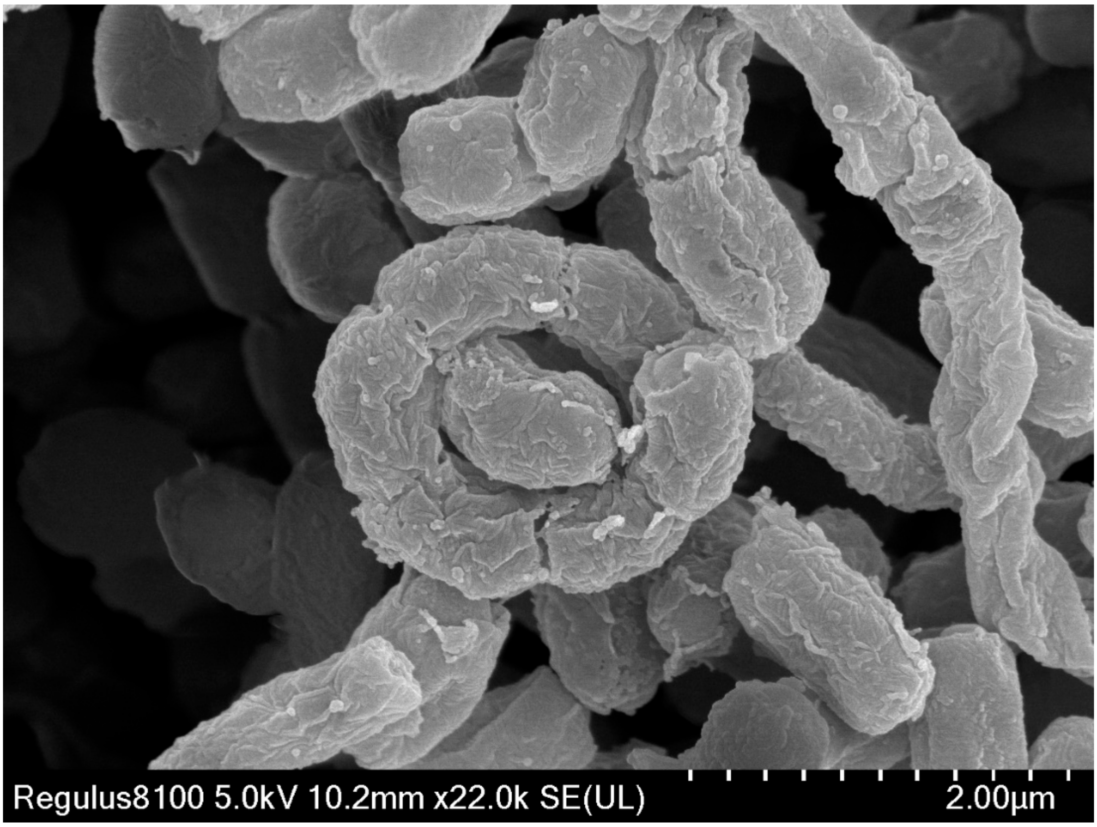


**Figure S2.** Morphological characteristics of strain SA61 on different media. Strain SA61 grew well on media ISP2, ISP3, ISP4, ISP5, ISP7 and nutrient agar (NA), and moderately on ISP1, ISP6, BA and CA media.

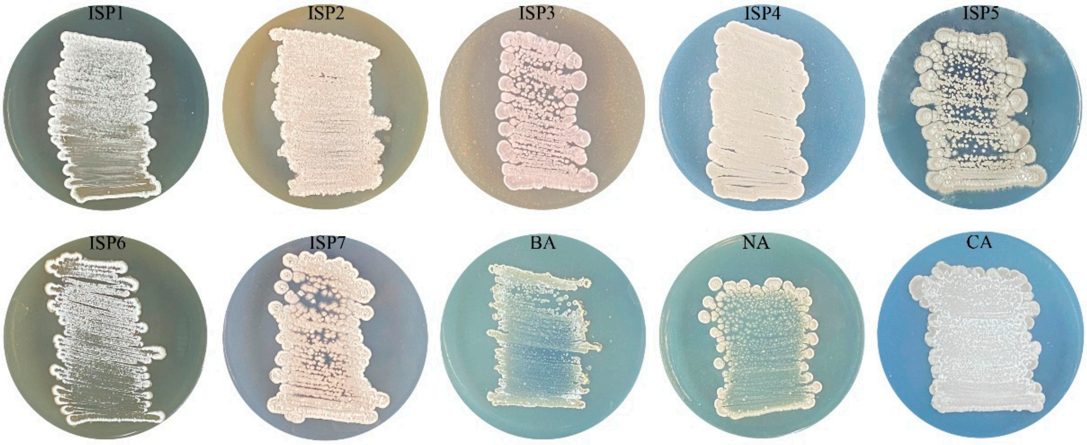

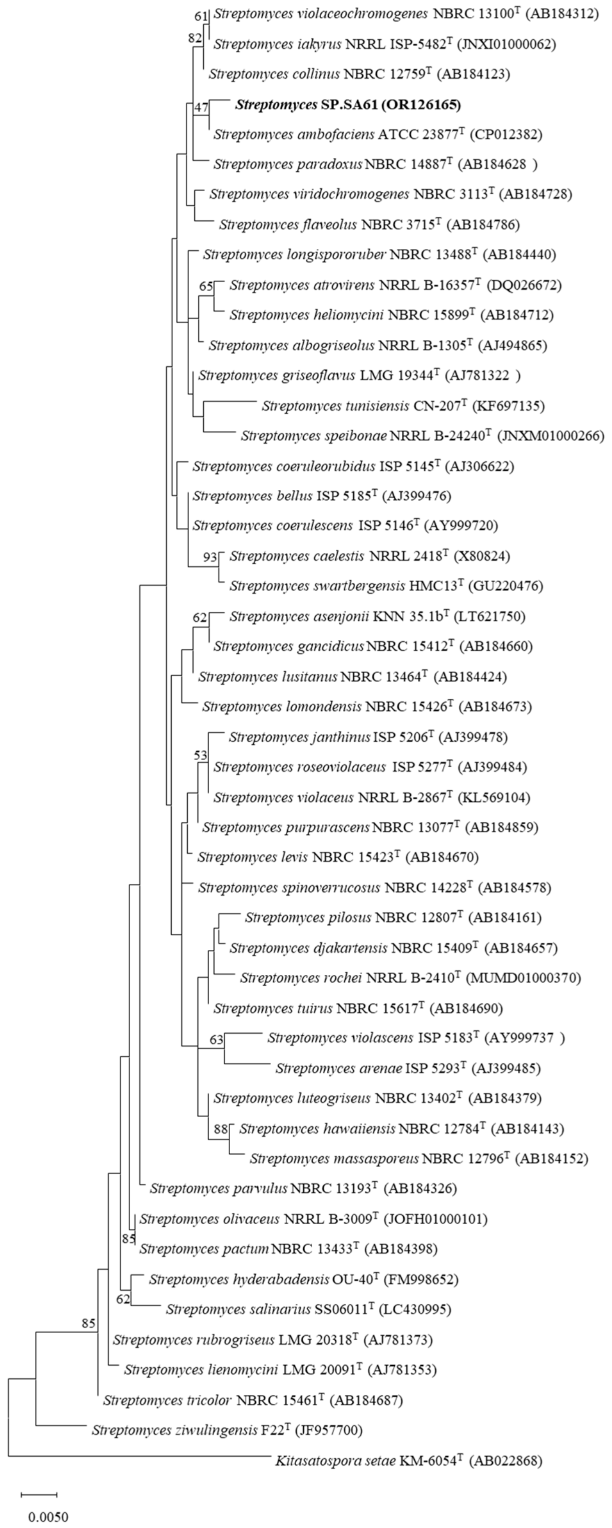


**Figure S3.** Neighbour-joining phylogenetic tree based on the 16S rRNA gene sequences with members of the genus *Streptomyces*. Bootstrap values (expressed as percentages of 1000 replications) of above 50 % are shown at branch points. GenBank accession numbers are given in parentheses. *Kitasatospora setae* KM-6054$^{T}$ was used as the outgroup. Bar, 0.005 substitution per nucleotide position.

**Figure S4.** The $^{1}$H-NMR spectrum of compound **1** in $CDCl_3$

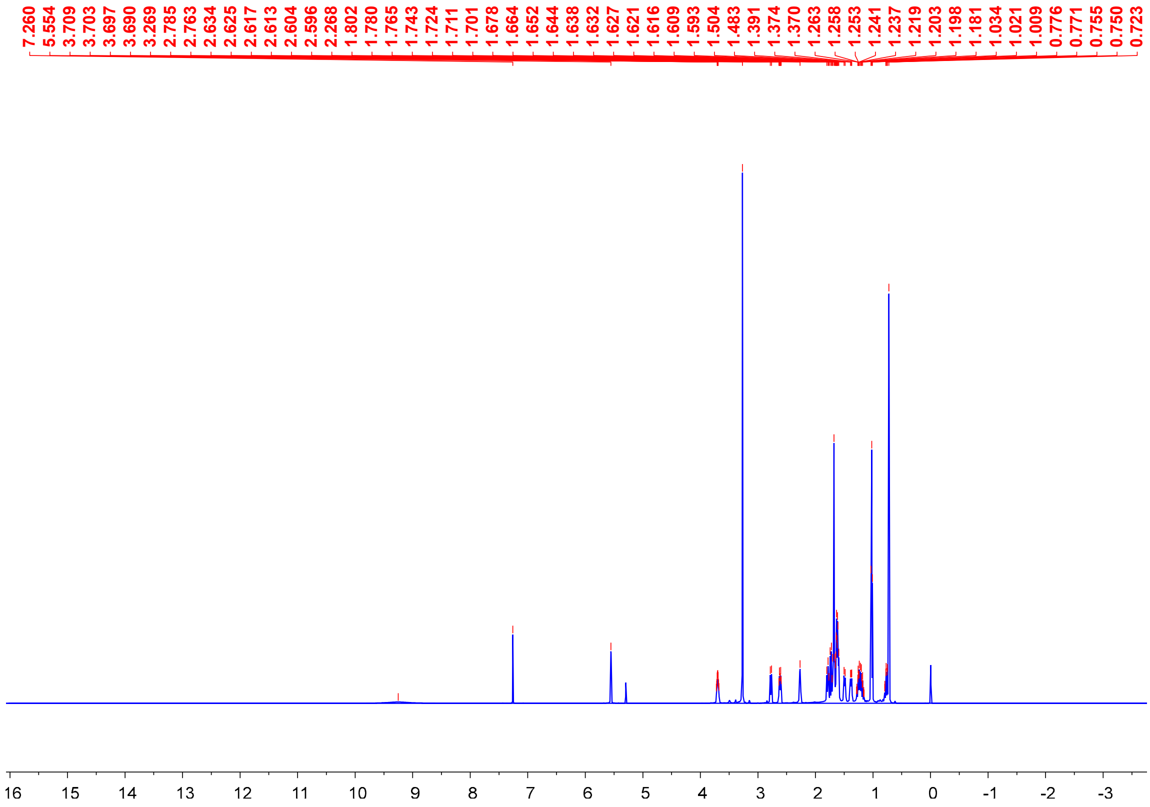


**Figure S5.** The $^{13}$C-NMR spectrum of compound **1** in $CDCl_3$

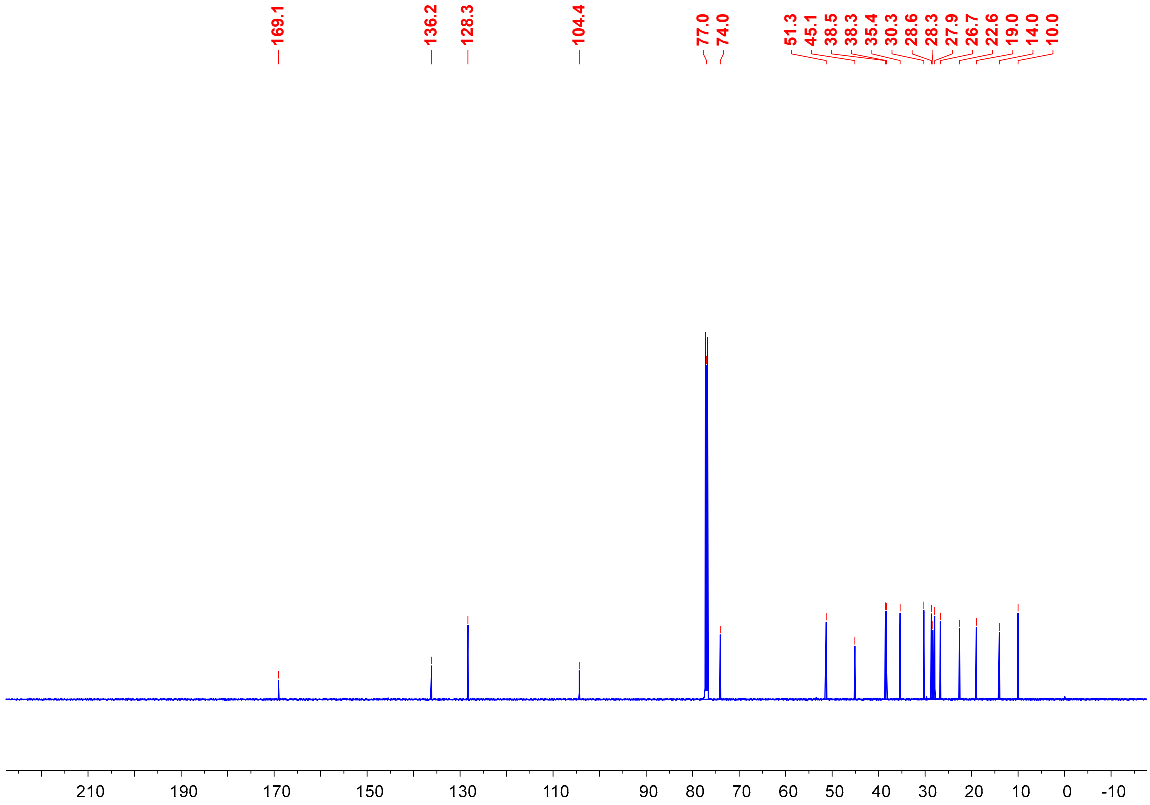

**Figure S6.** The HSQC spectrum of compound **1** in $CDCl_3$

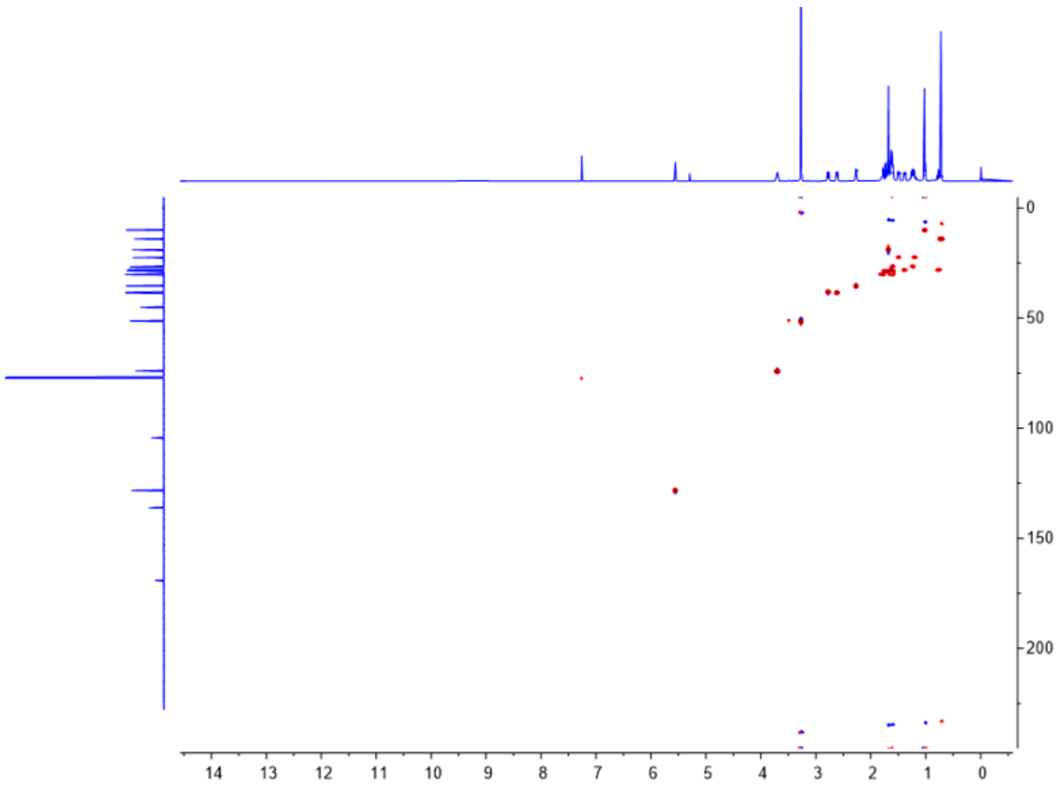


**Figure S7.** The HMBC spectrum of compound **1** in $CDCl_3$

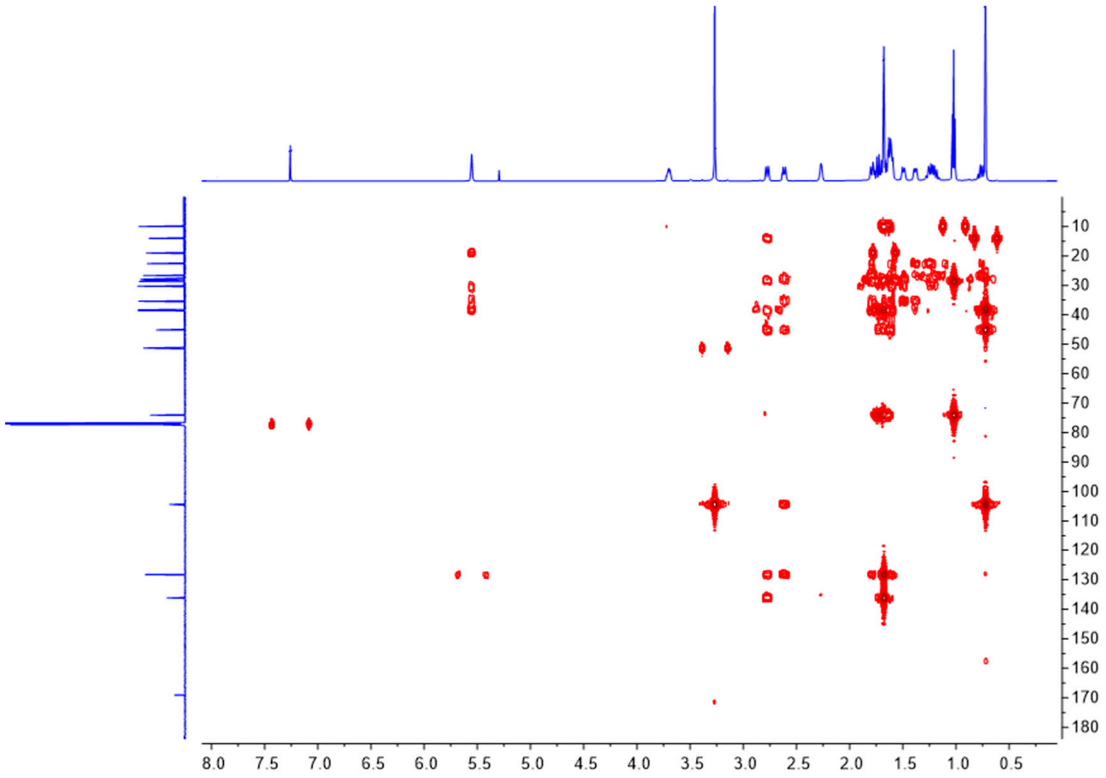

**Figure S8.** The $^{1}H$-$^{1}H$ COSY spectrum of compound **1** in $CDCl_3$

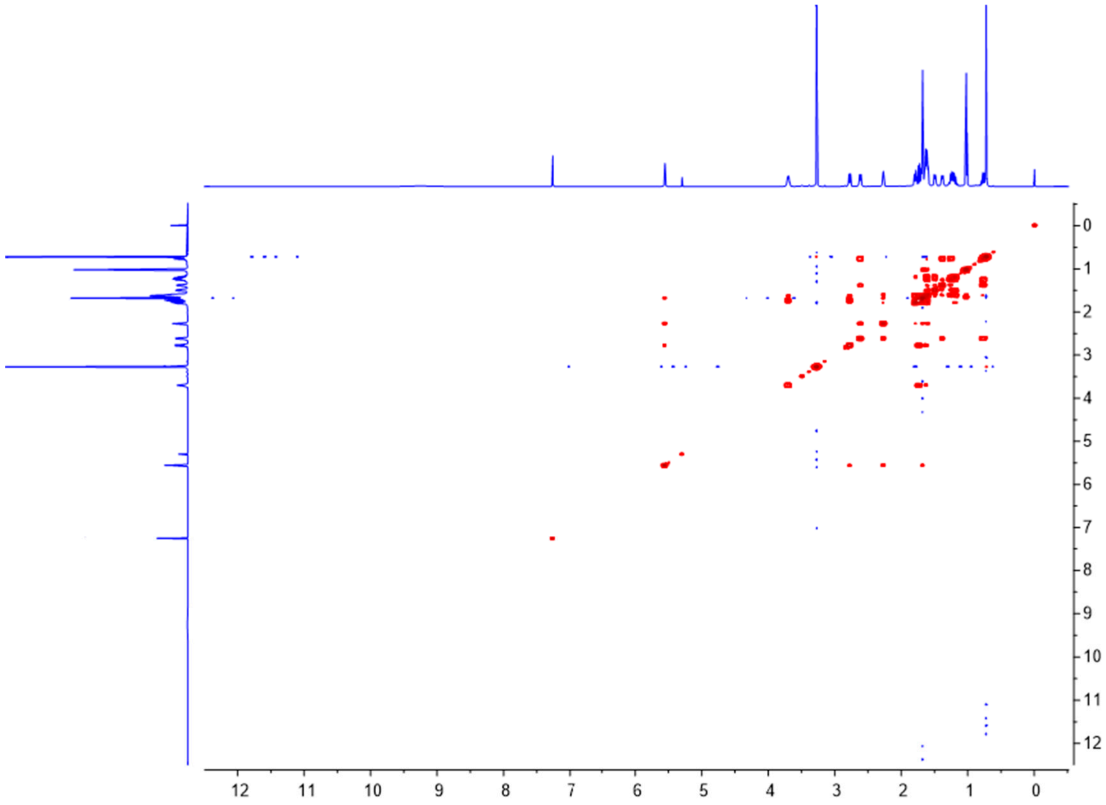


**Figure S9.** The NOESY spectrum of compound **1** in $CDCl_3$

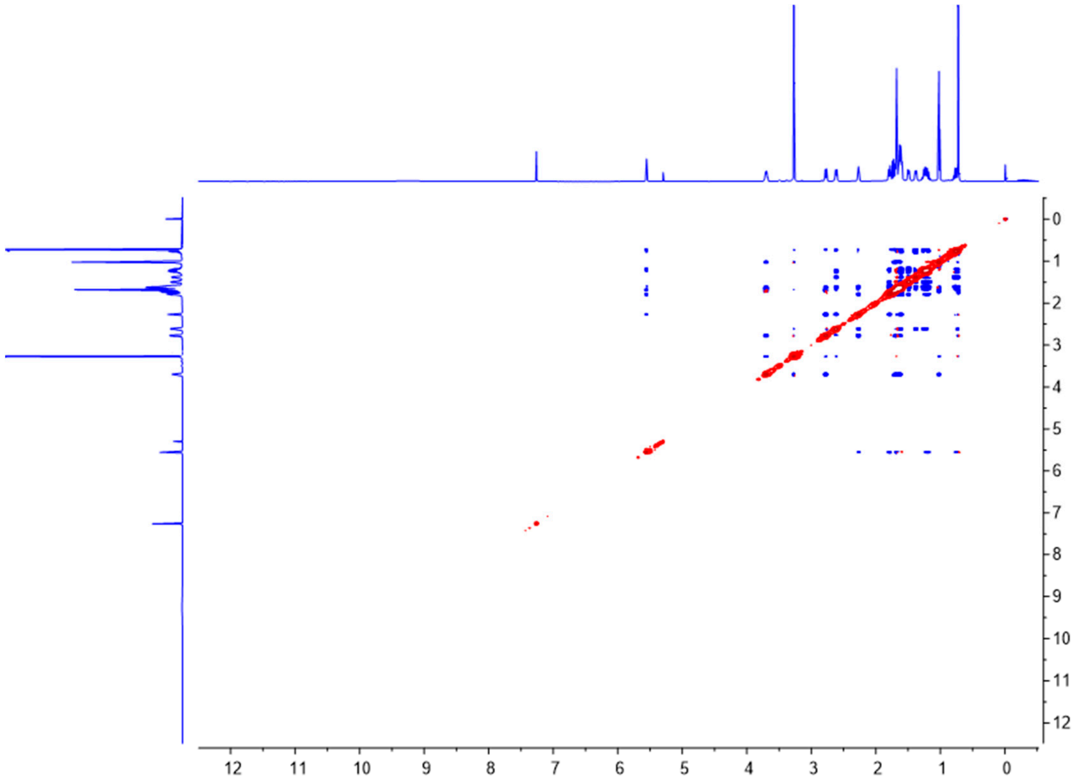

**Figure S10.** The HRESIMS spectrum of compound **1**

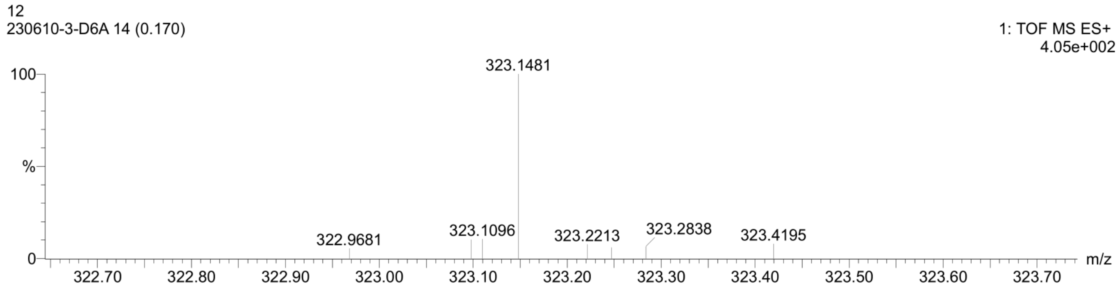


**Figure S11.** The $^{1}$H-NMR spectrum of compound **2** in $CDCl_3$

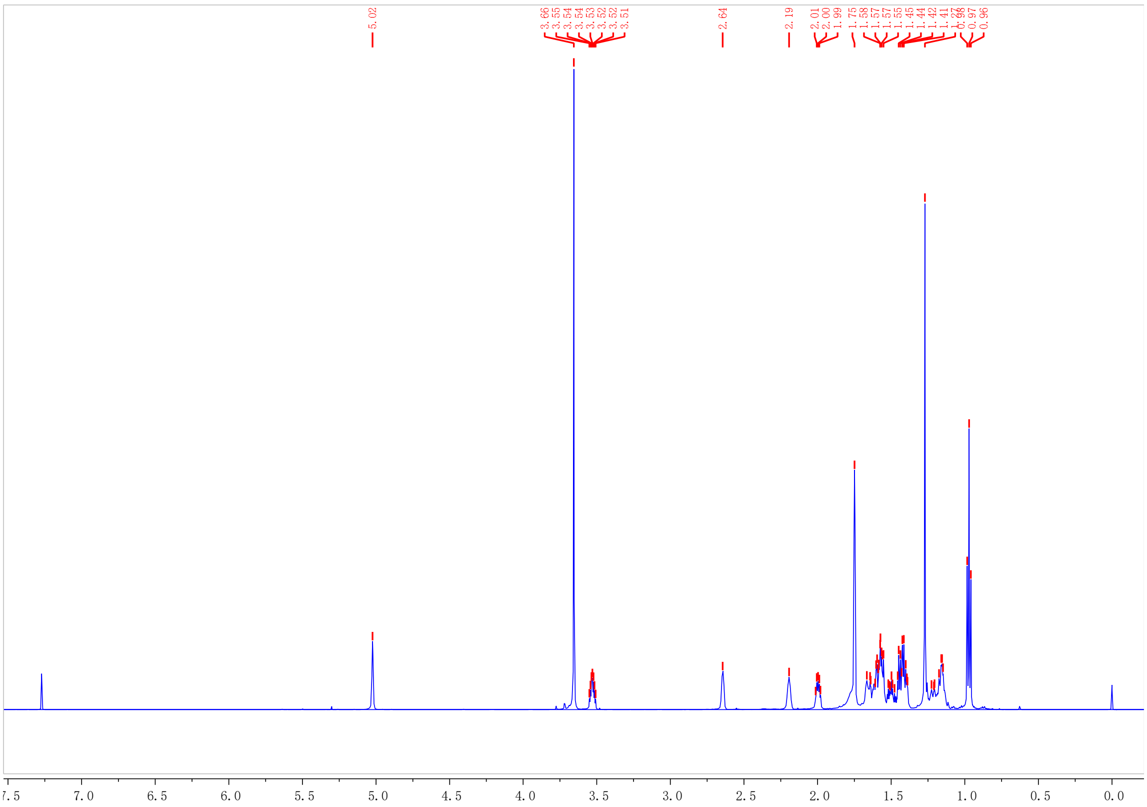

**Figure S12.** The $^{13}C$-NMR spectrum of compound **2** in $CDCl_3$

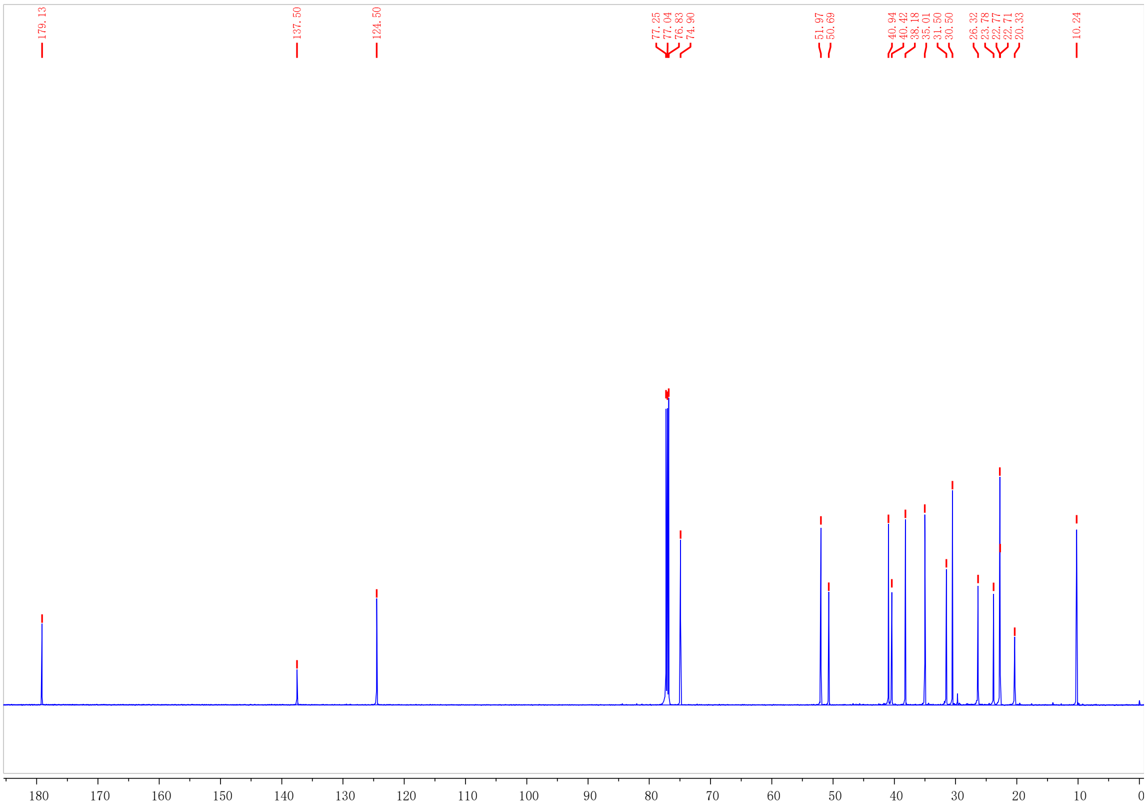


**Figure S13.** The HSQC spectrum of compound **2** in $CDCl_3$

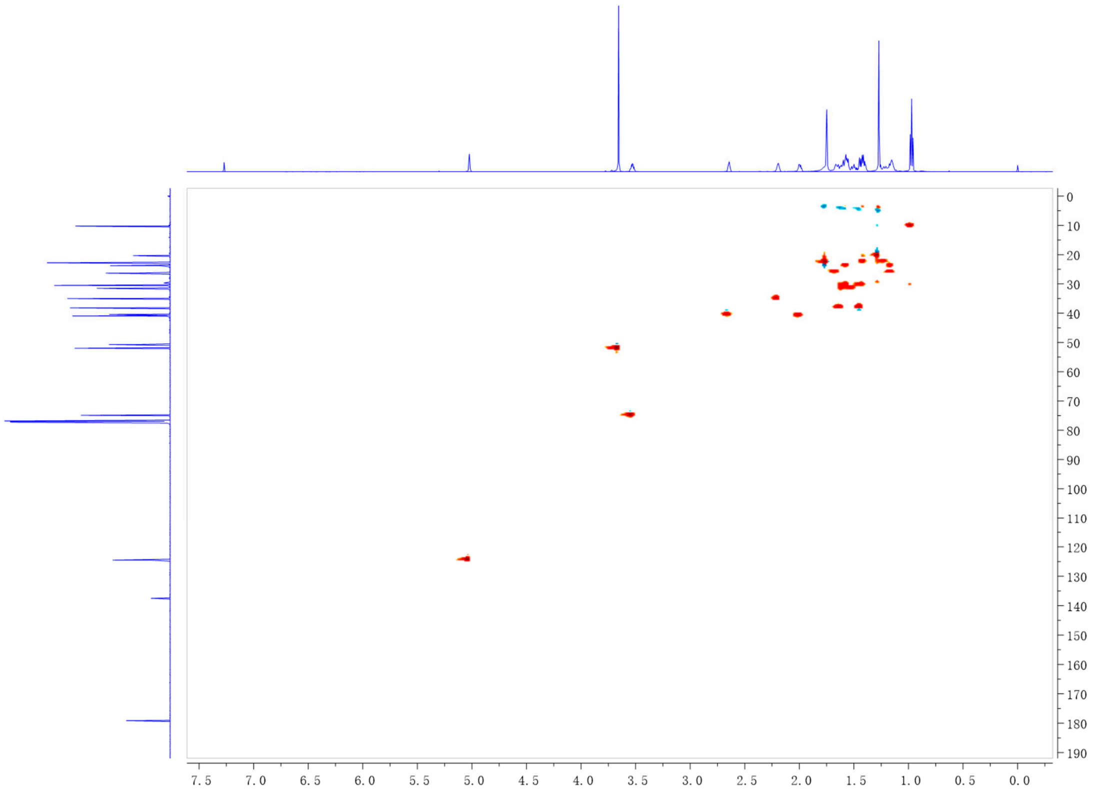

**Figure S14.** The HMBC spectrum of compound **2** in $CDCl_3$

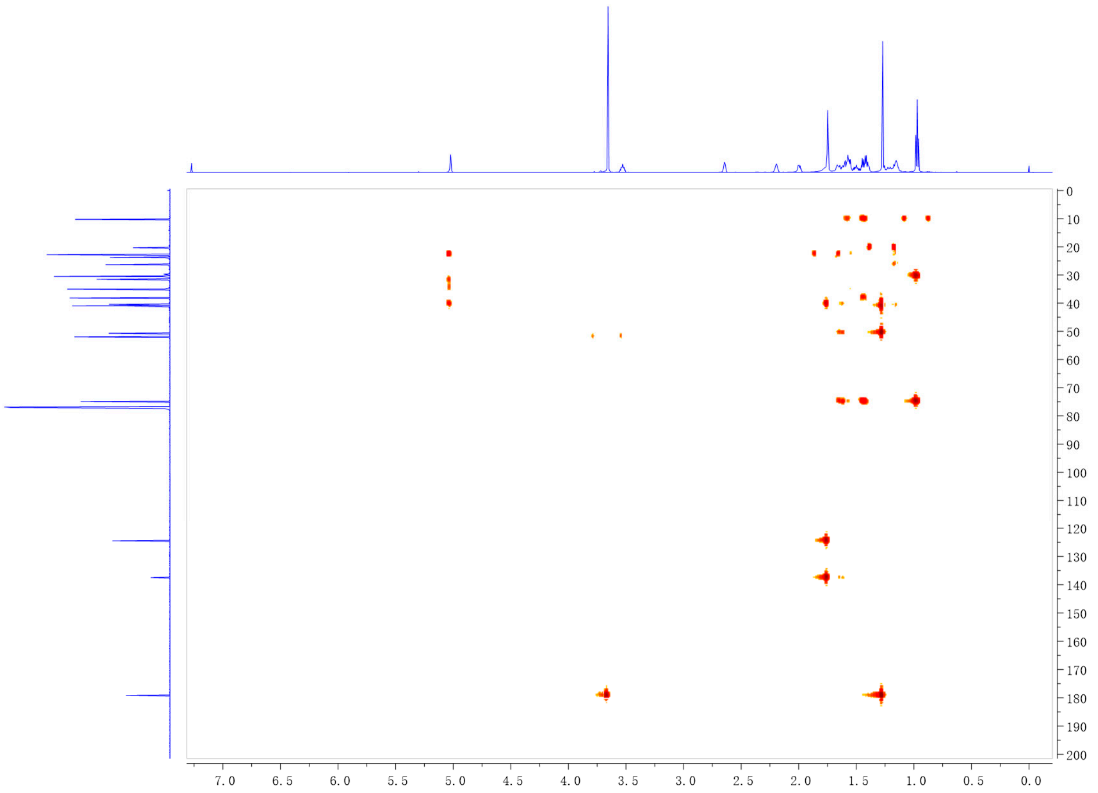


**Figure S15.** The $^1H$-$^1H$ COSY spectrum of compound **2** in $CDCl_3$

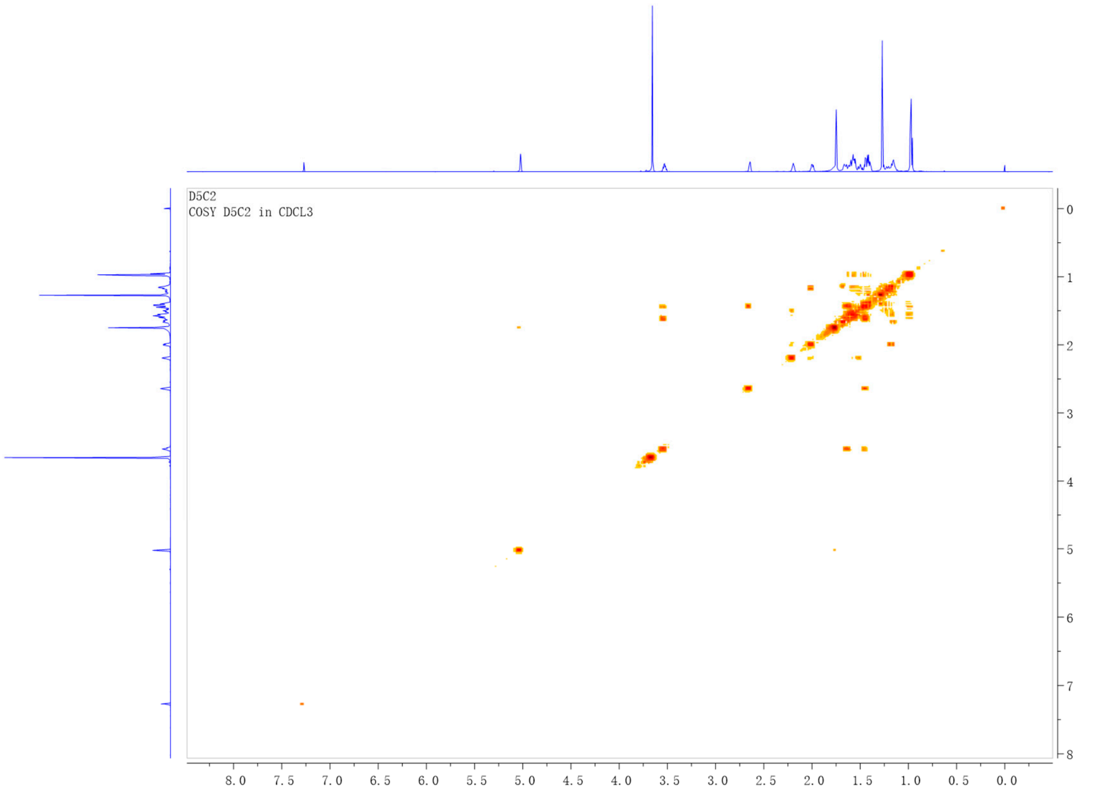

**Figure S16.** The NOESY spectrum of compound **2** in $CDCl_3$

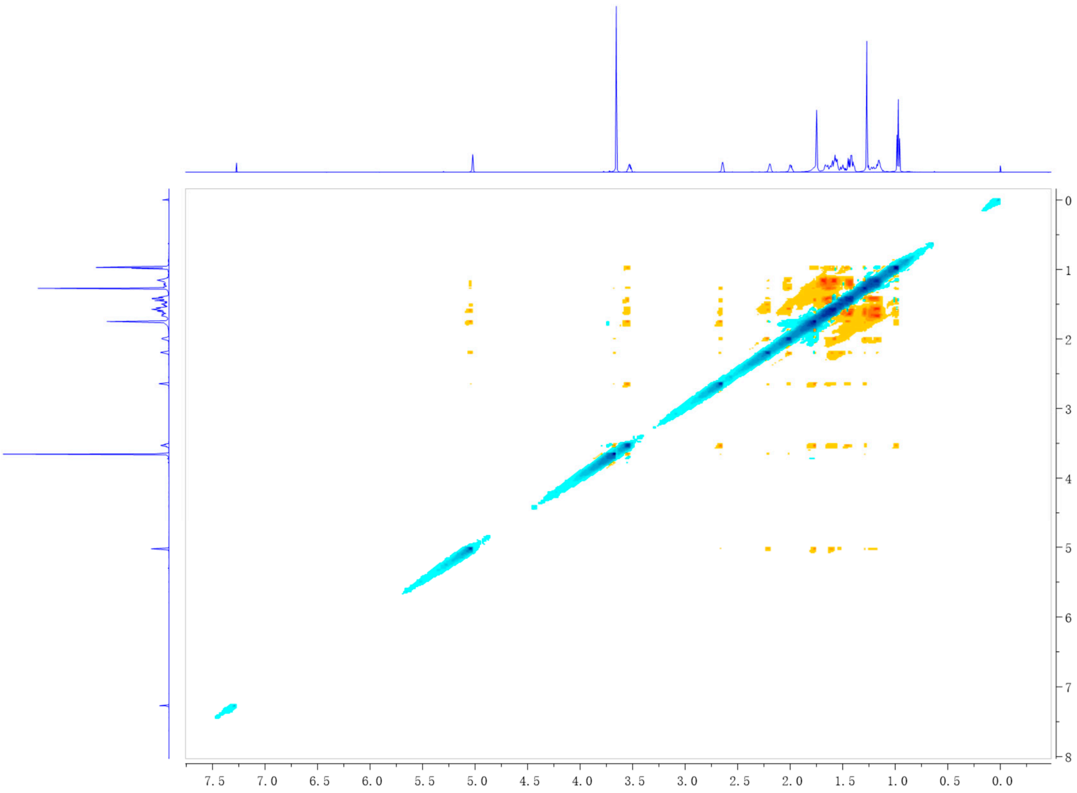


**Figure S17.** The HRESIMS spectrum of compound **2**

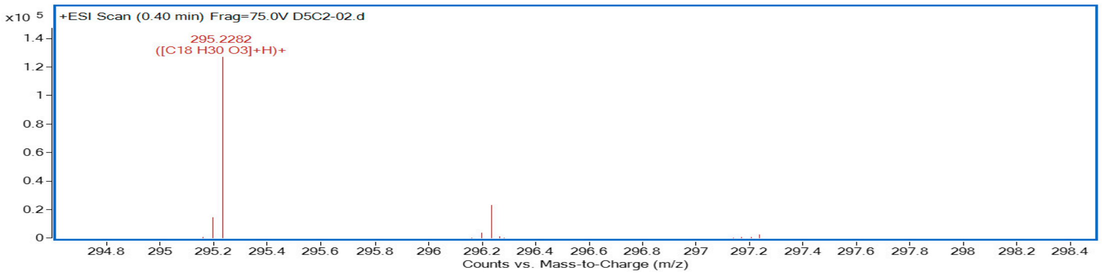

**Figure S18.** The $^{1}$H-NMR spectrum of compound **3** in $CDCl_3$

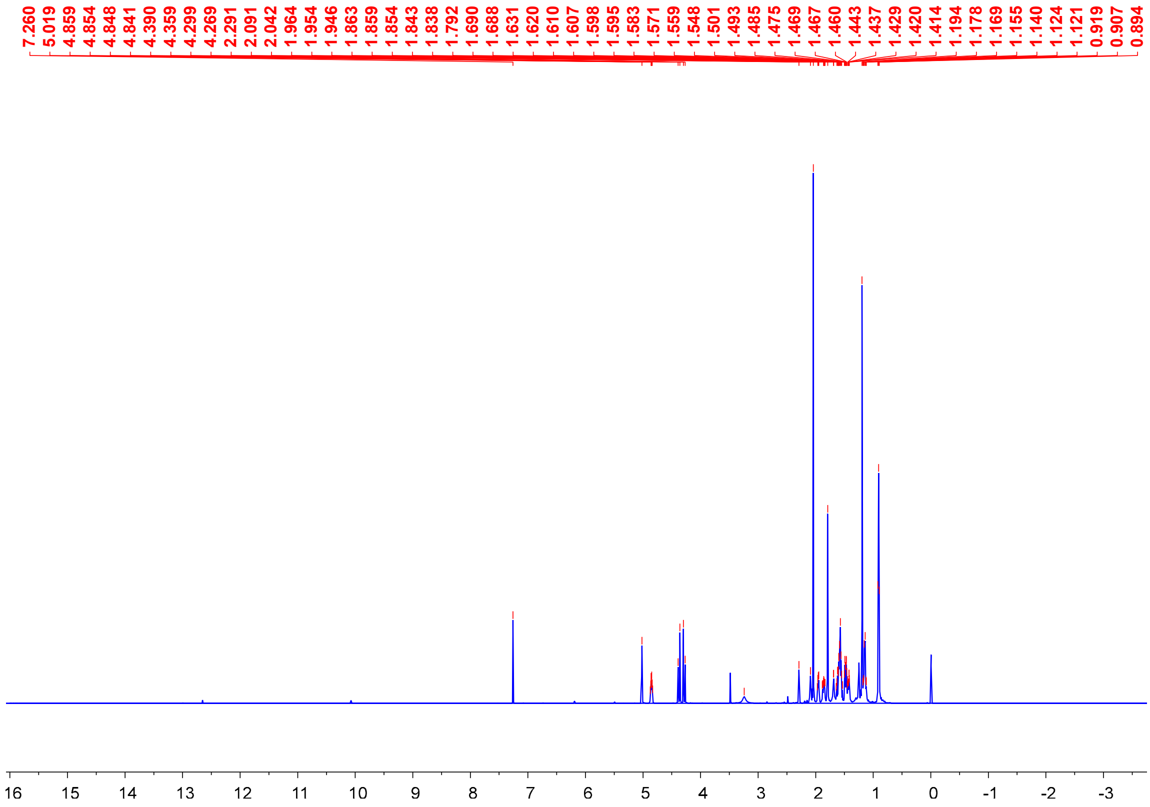

**Figure S19.** The $^{13}$C-NMR spectrum of compound **3** in $CDCl_3$

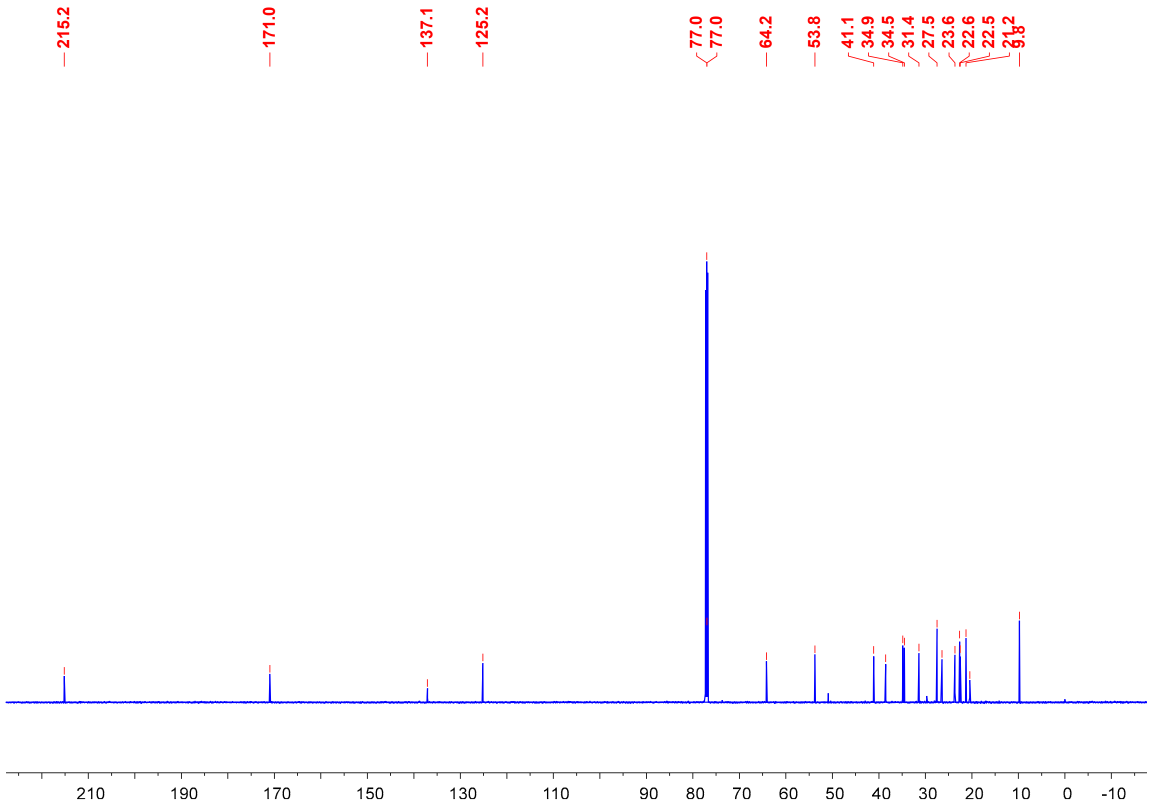

**Figure S20.** The HSQC spectrum of compound **3** in $CDCl_3$

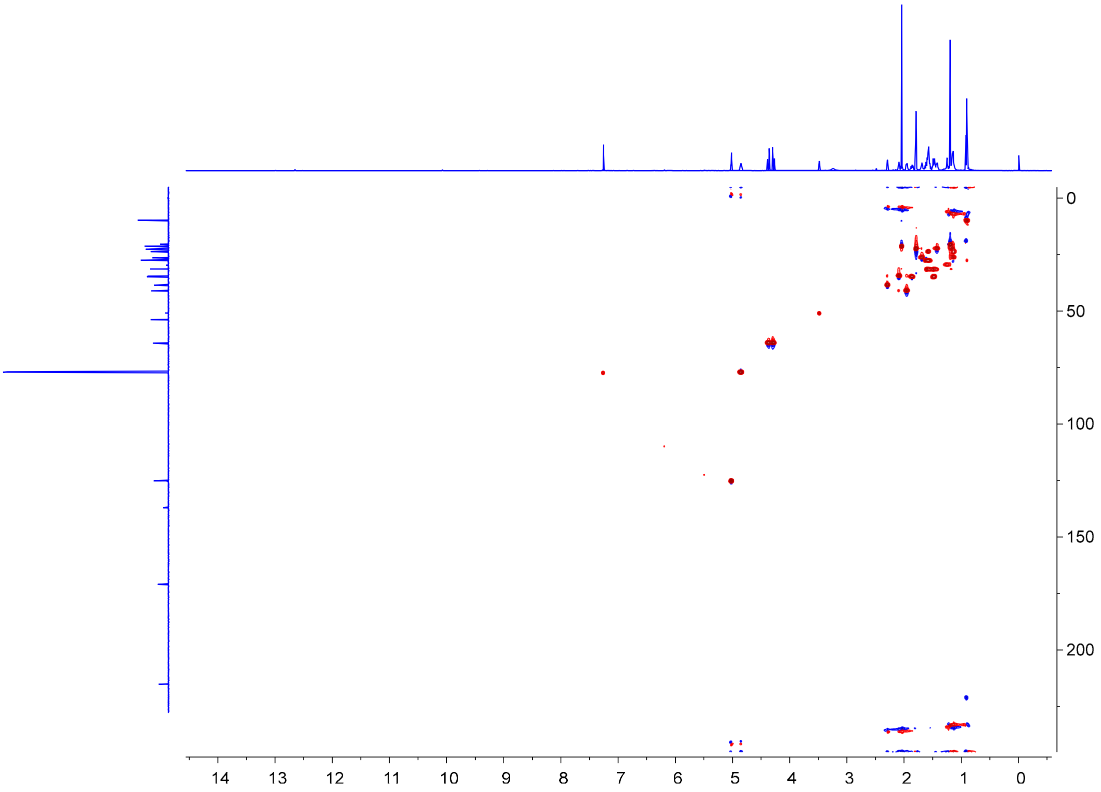


**Figure S21.** The HMBC spectrum of compound **3** in $CDCl_3$

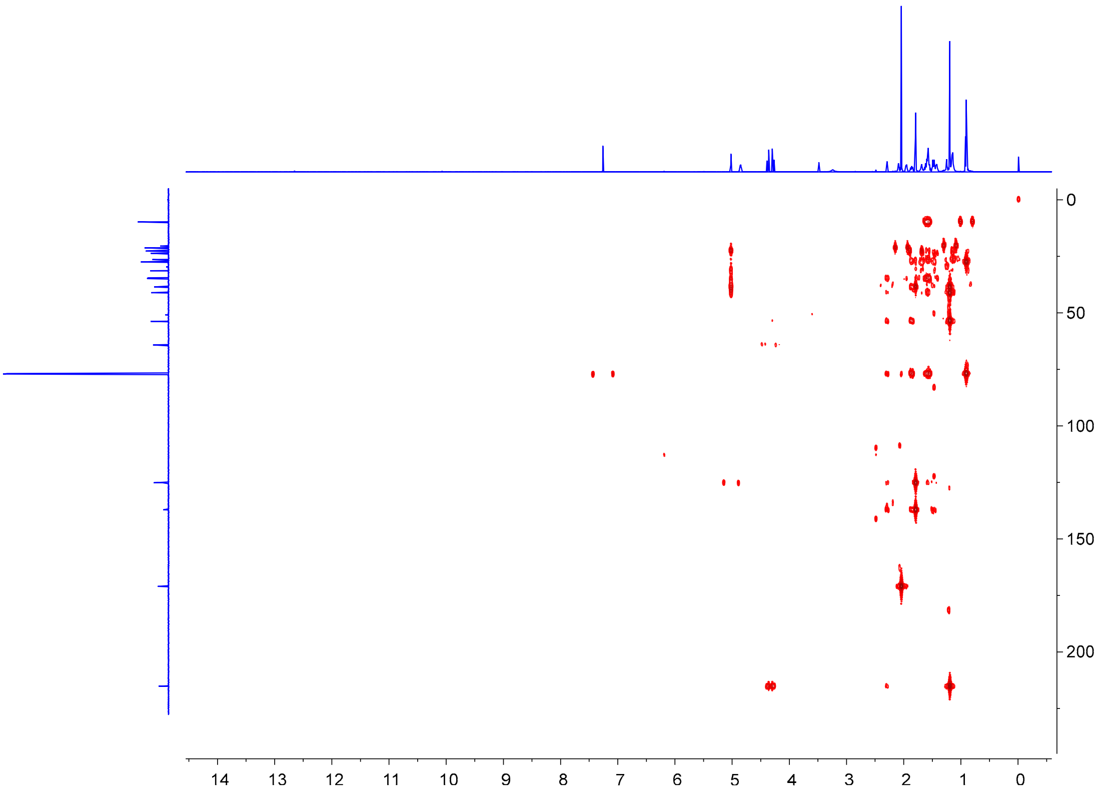

**Figure S22.** The $^{1}H$-$^{1}H$ COSY spectrum of compound **3** in $CDCl_3$

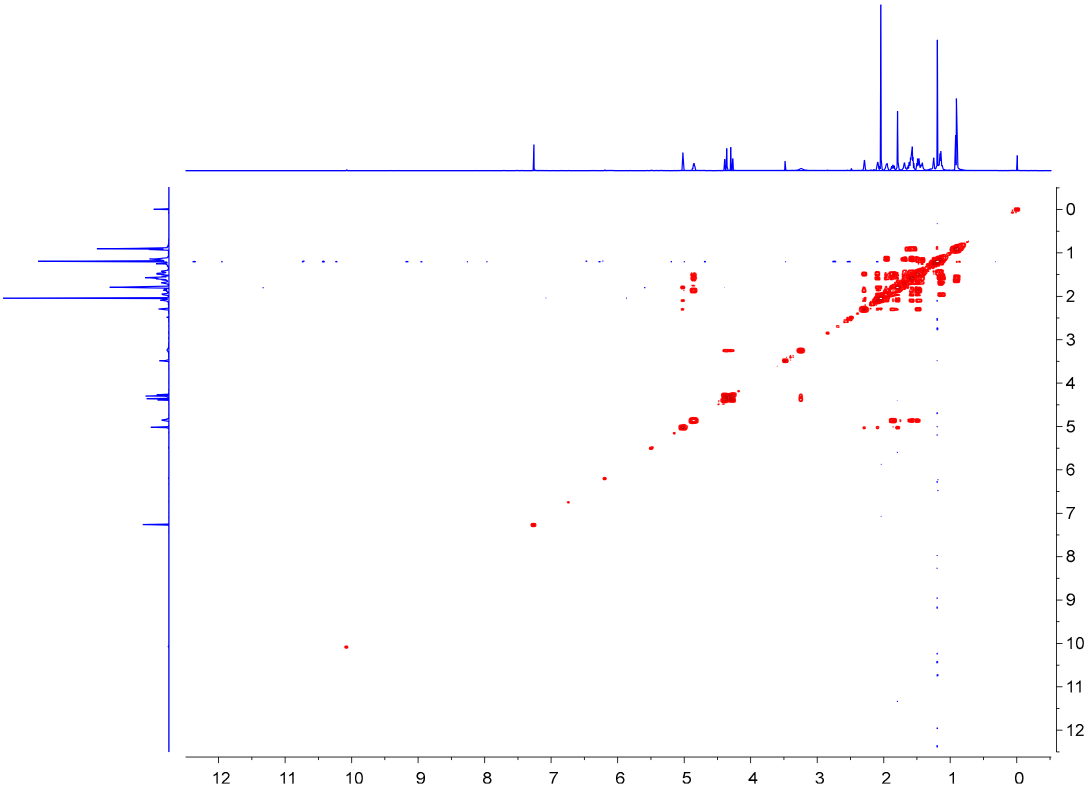


**Figure S23.** The NOESY spectrum of compound **3** in $CDCl_3$

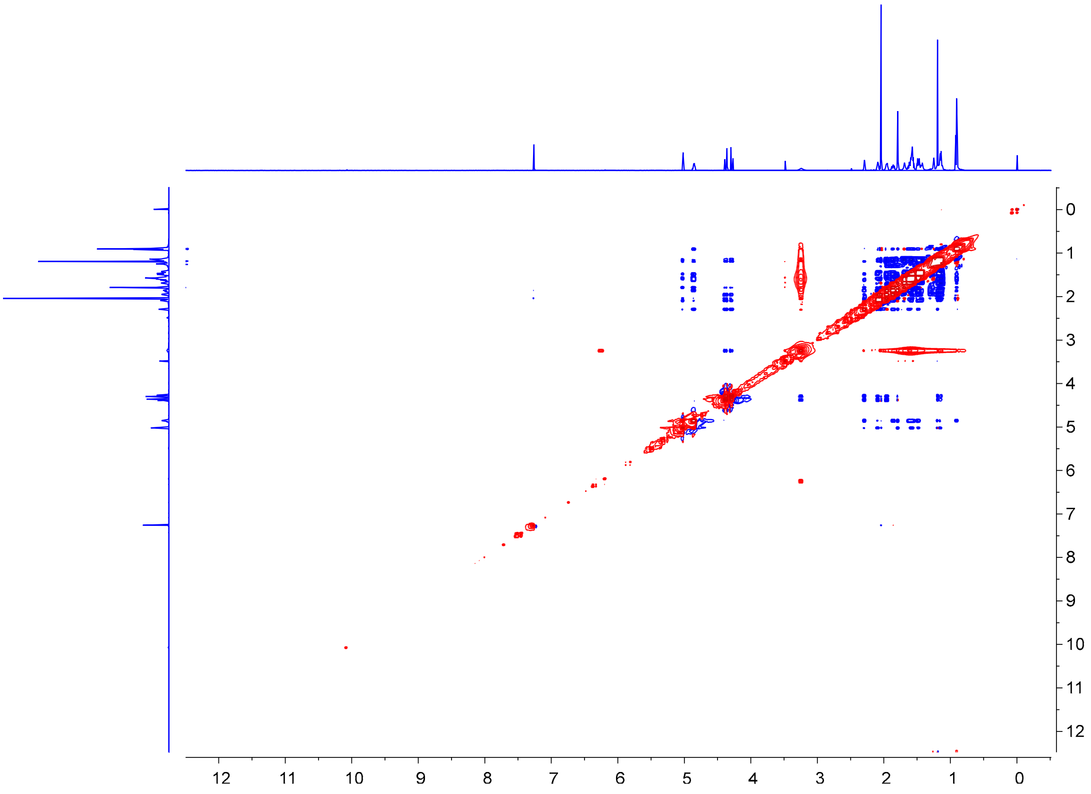

**Figure S24.** The HRESIMS spectrum of compound **3**

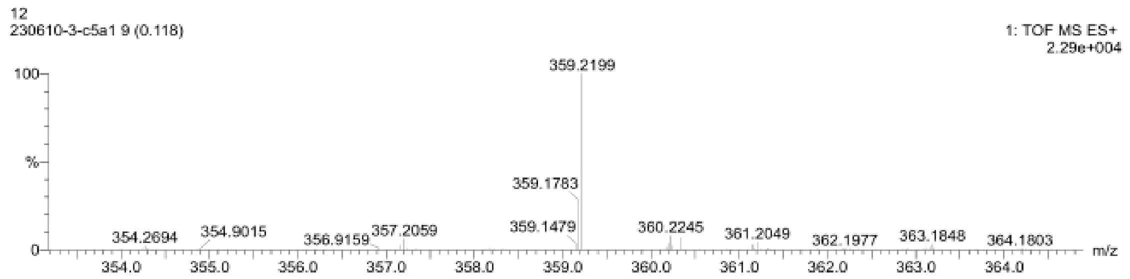


**Figure S25.** The $^{1}H$-NMR spectrum of compound **4** in $CDCl_3$

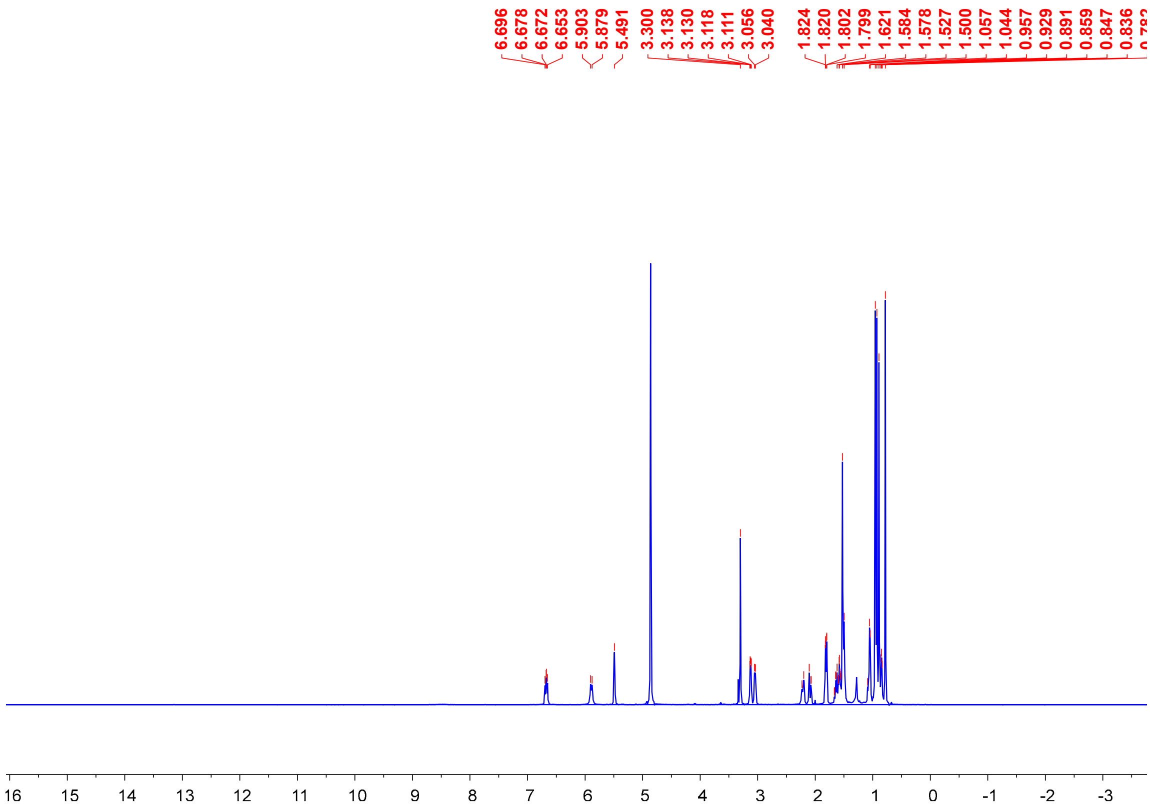

**Figure S26.** The $^{13}$C-NMR spectrum of compound **4** in $CDCl_3$

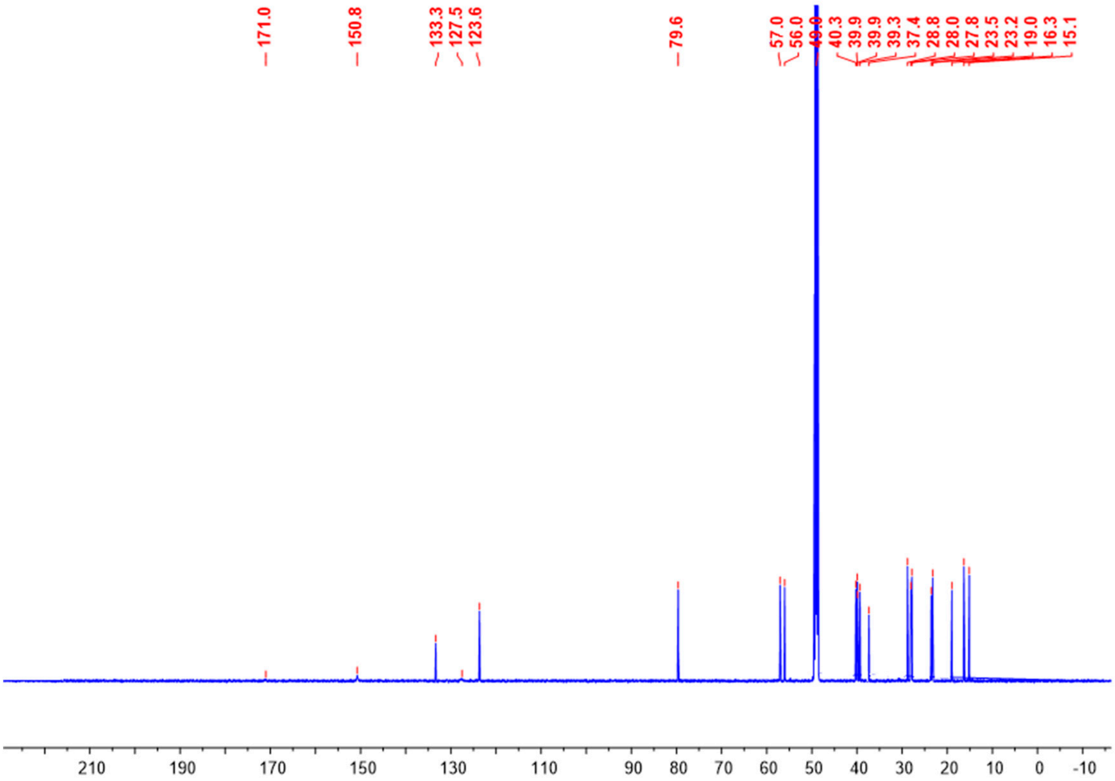


**Figure S27.** The HSQC spectrum of compound **4** in $CDCl_3$

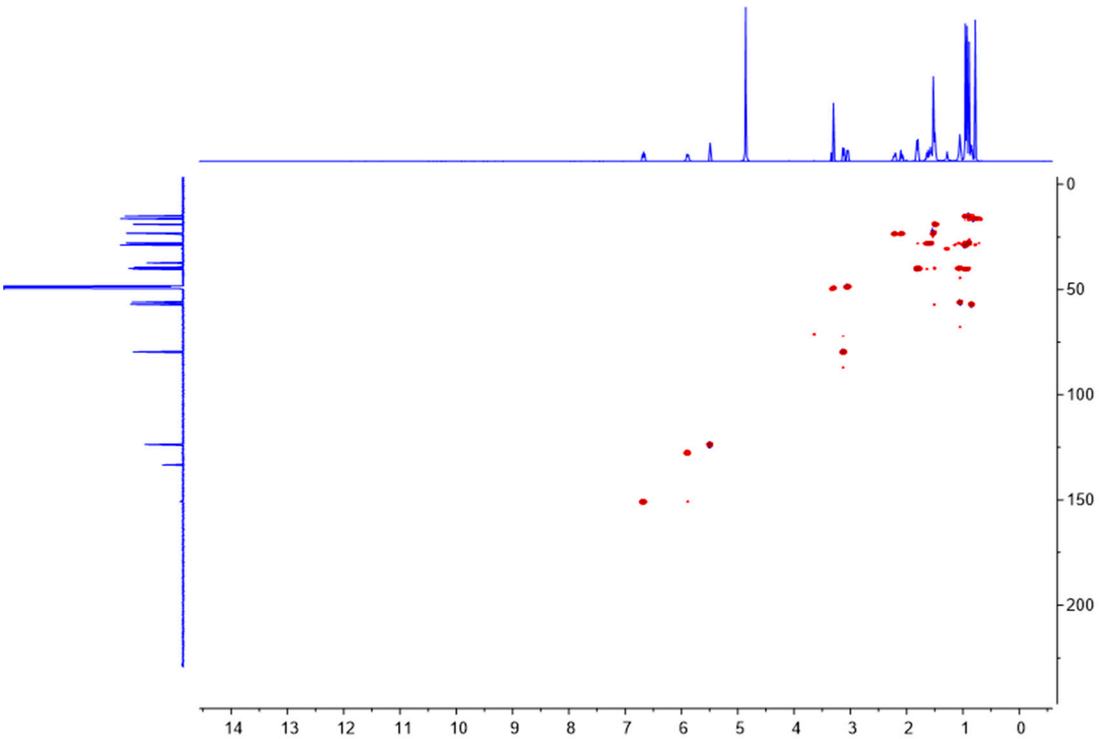

**Figure S28.** The HMBC spectrum of compound **4** in $CDCl_3$

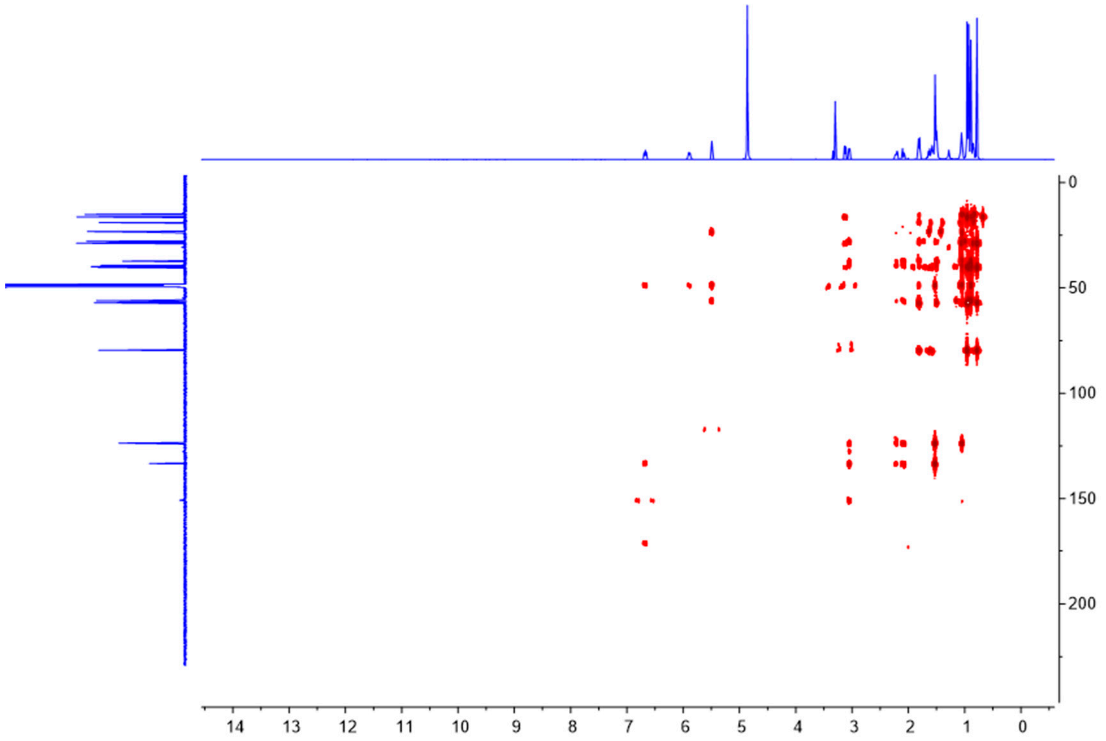


**Figure S29.** The $^1H$-$^1H$ COSY spectrum of compound **4** in $CDCl_3$

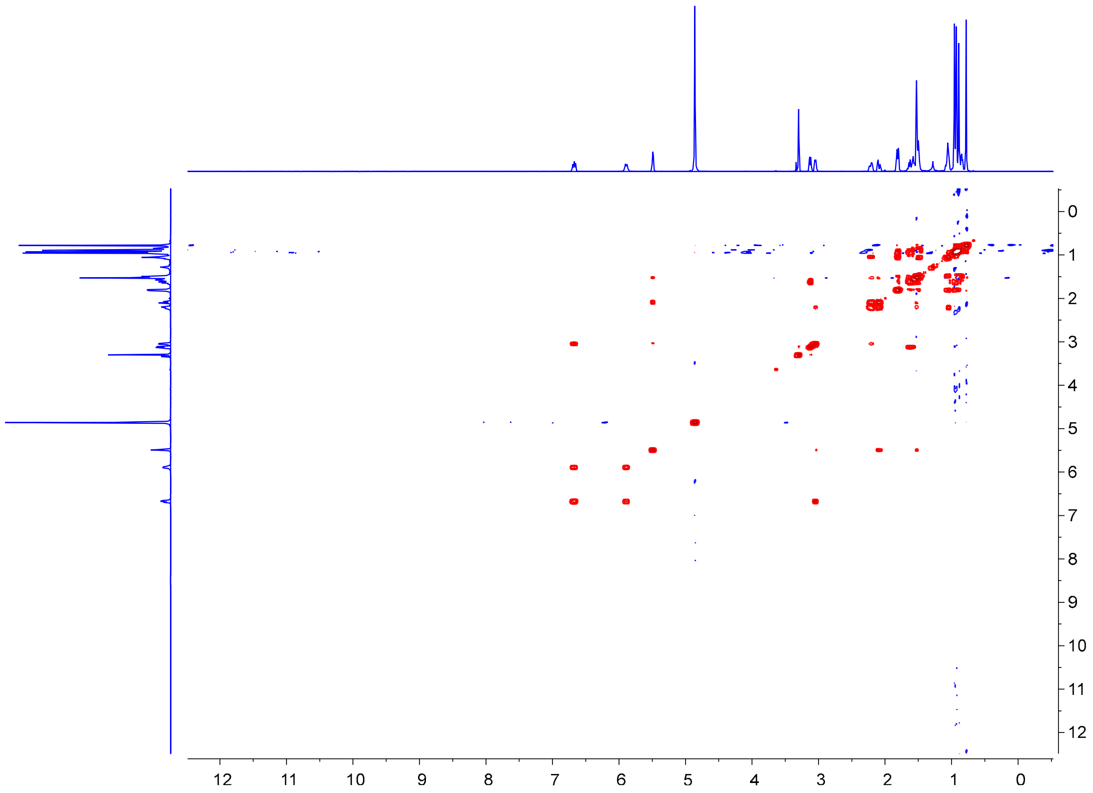

**Figure S30.** The NOESY spectrum of compound **4** in $CDCl_3$

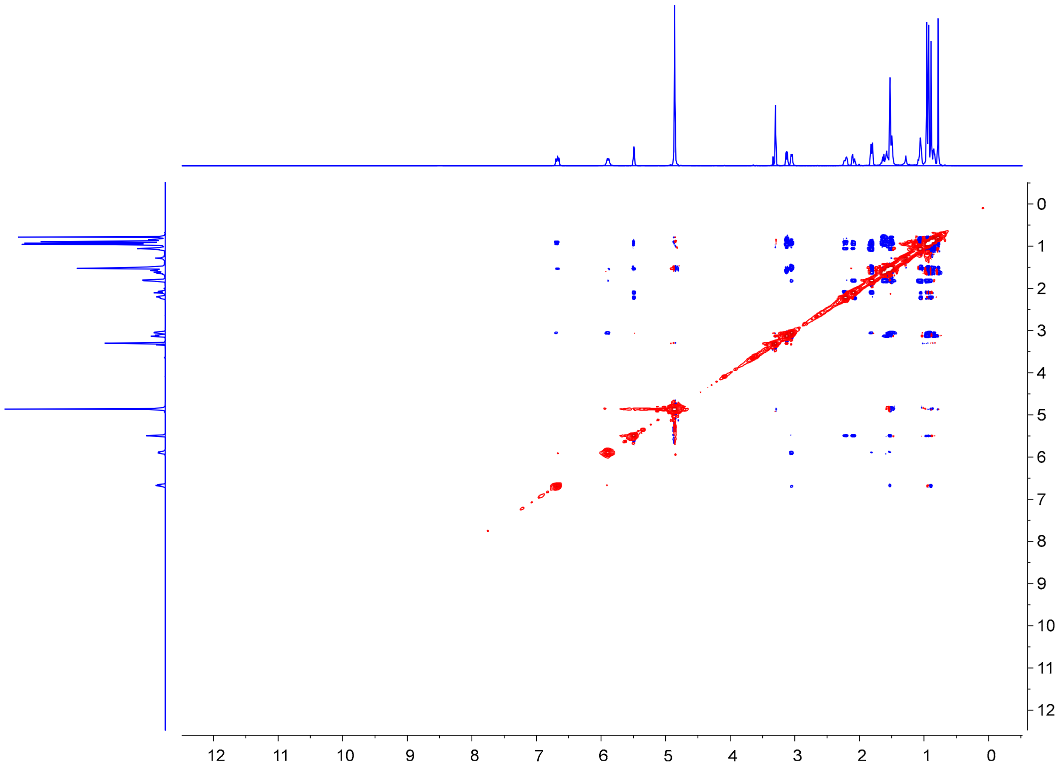

**Figure S31.** The HRESIMS spectrum of compound **4**

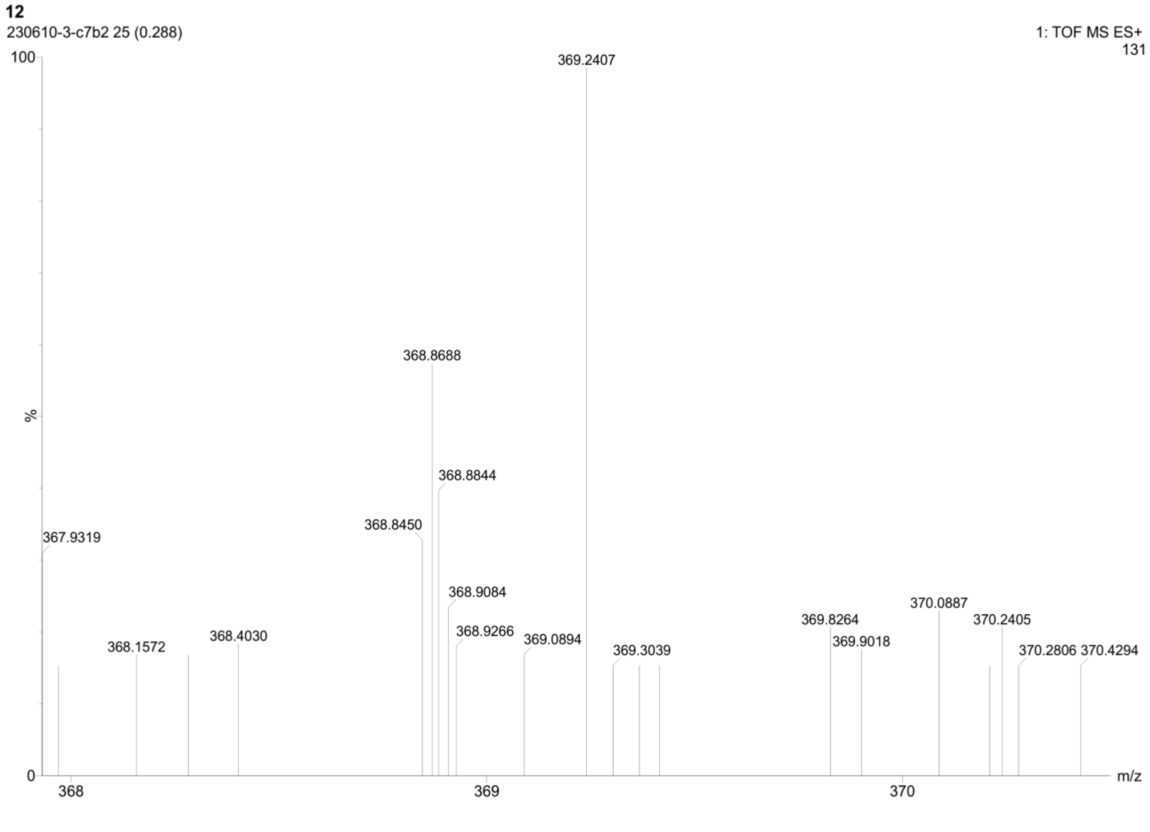

**Figure S32.** The $^{1}$H-NMR spectrum of compound **5** in $CDCl_3$

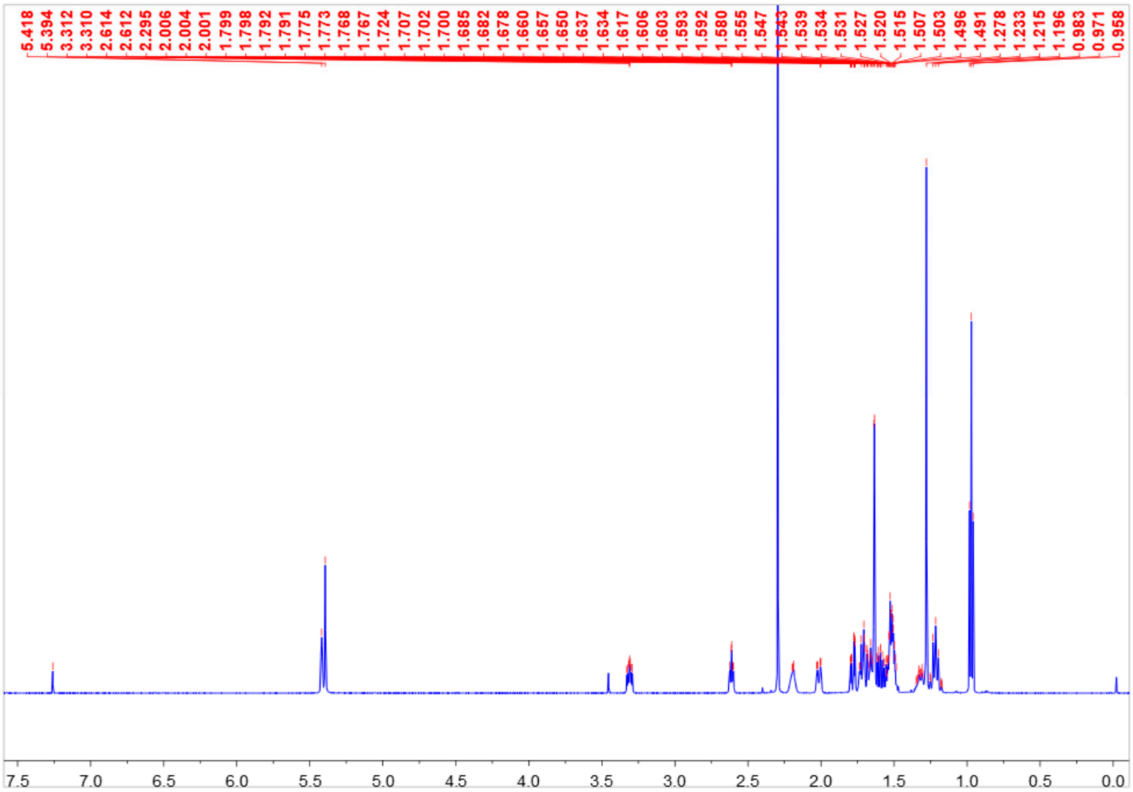


**Figure S33.** The $^{13}$C-NMR spectrum of compound **5** in $CDCl_3$

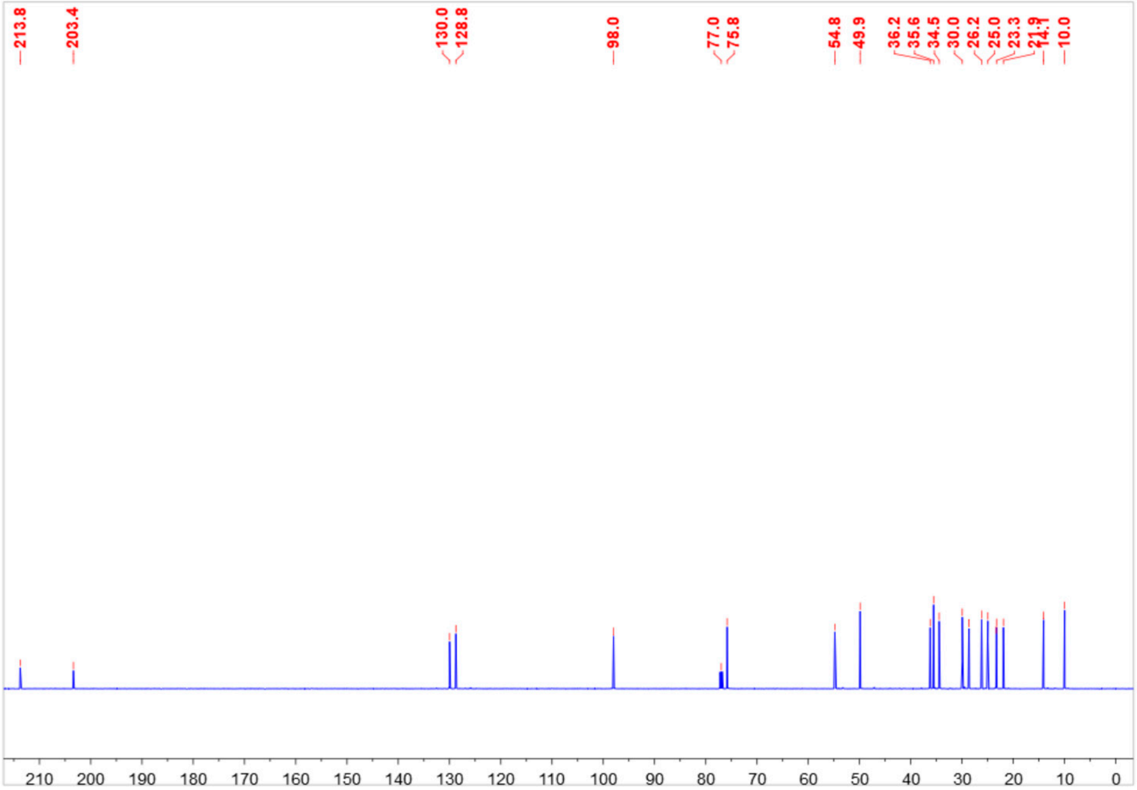

**Figure S34.** The HSQC spectrum of compound **5** in $CDCl_3$

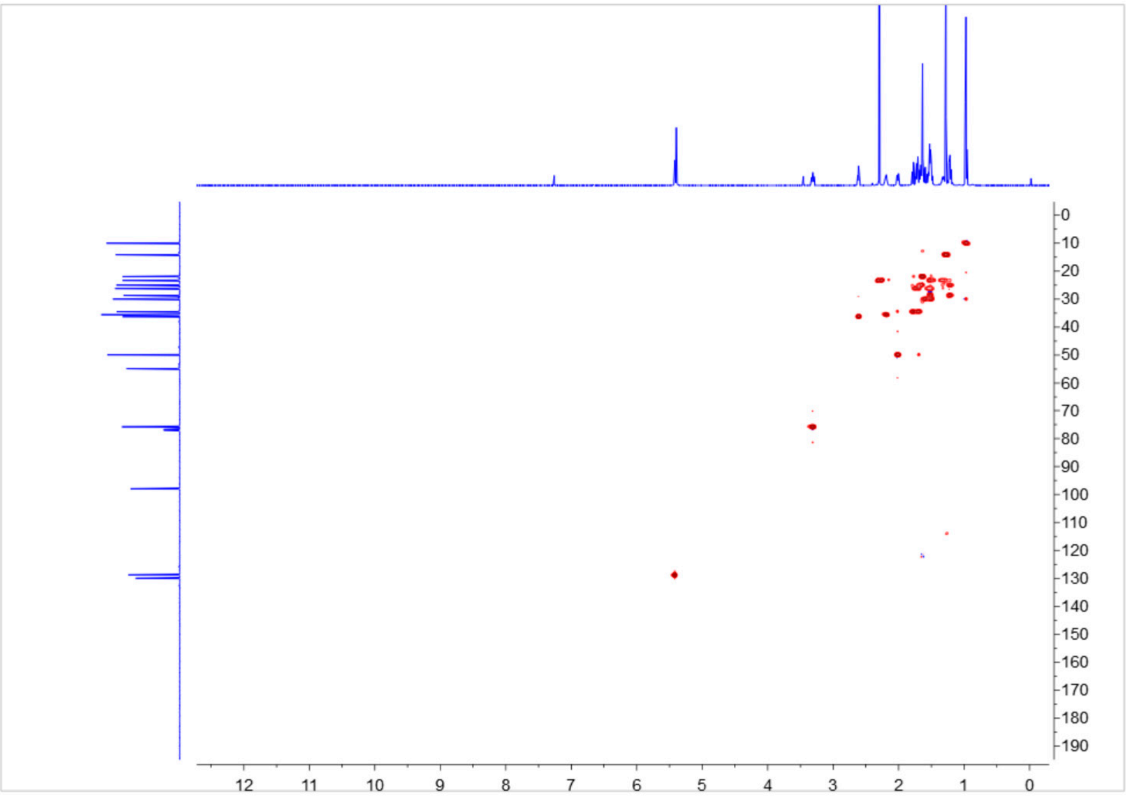


**Figure S35.** The HMBC spectrum of compound **5** in $CDCl_3$

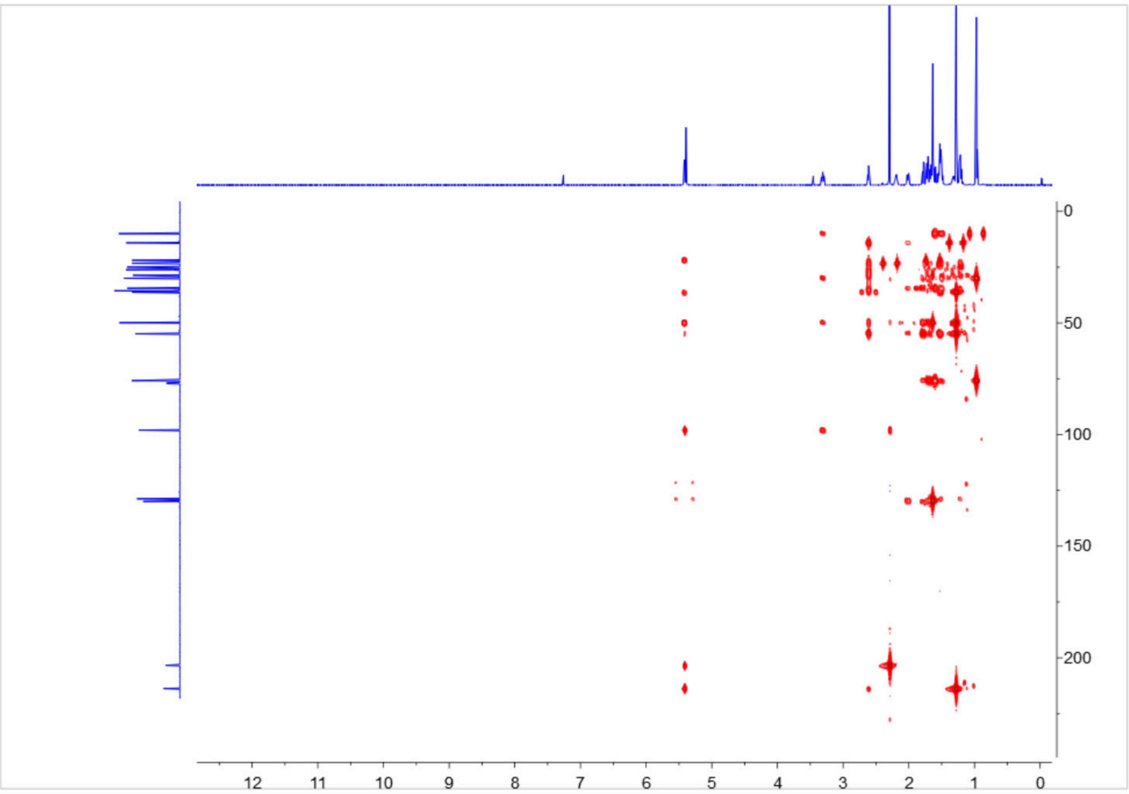

**Figure S36.** The $^{1}H$-$^{1}H$ COSY spectrum of compound **5** in $CDCl_3$

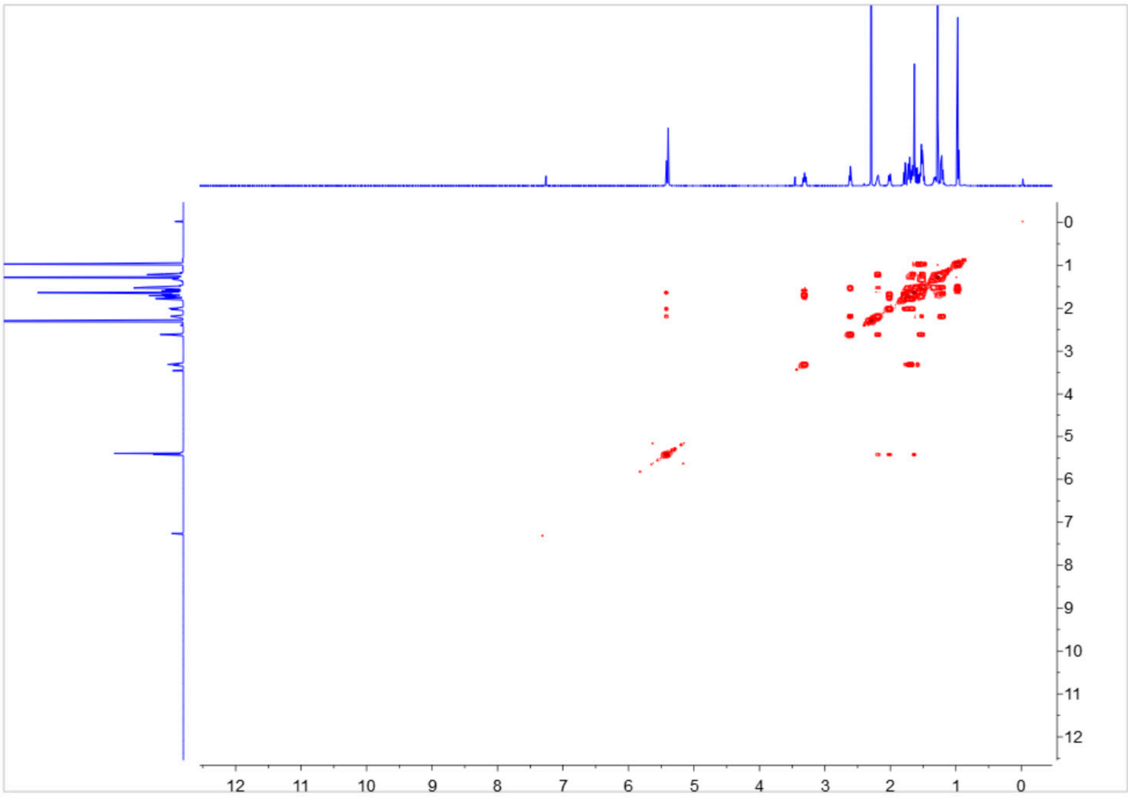


**Figure S37.** The NOESY spectrum of compound **5** in $CDCl_3$

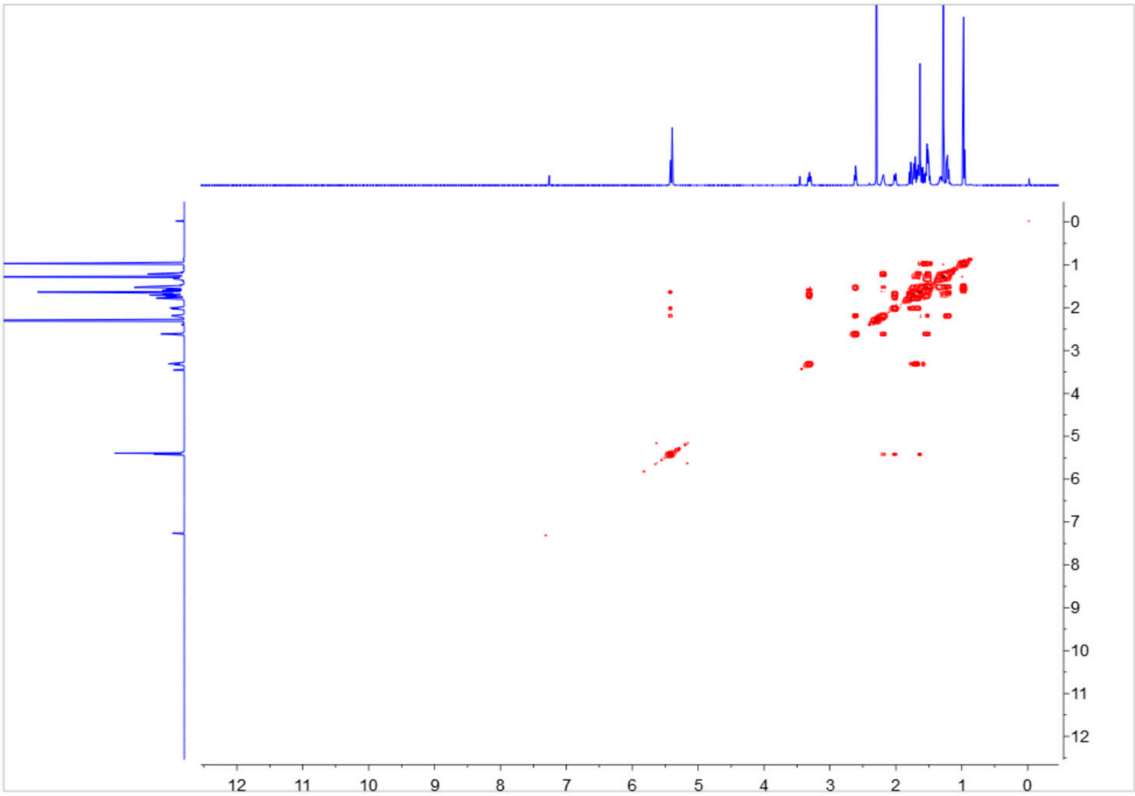

**Figure S38.** The HRESIMS spectrum of compound **5**

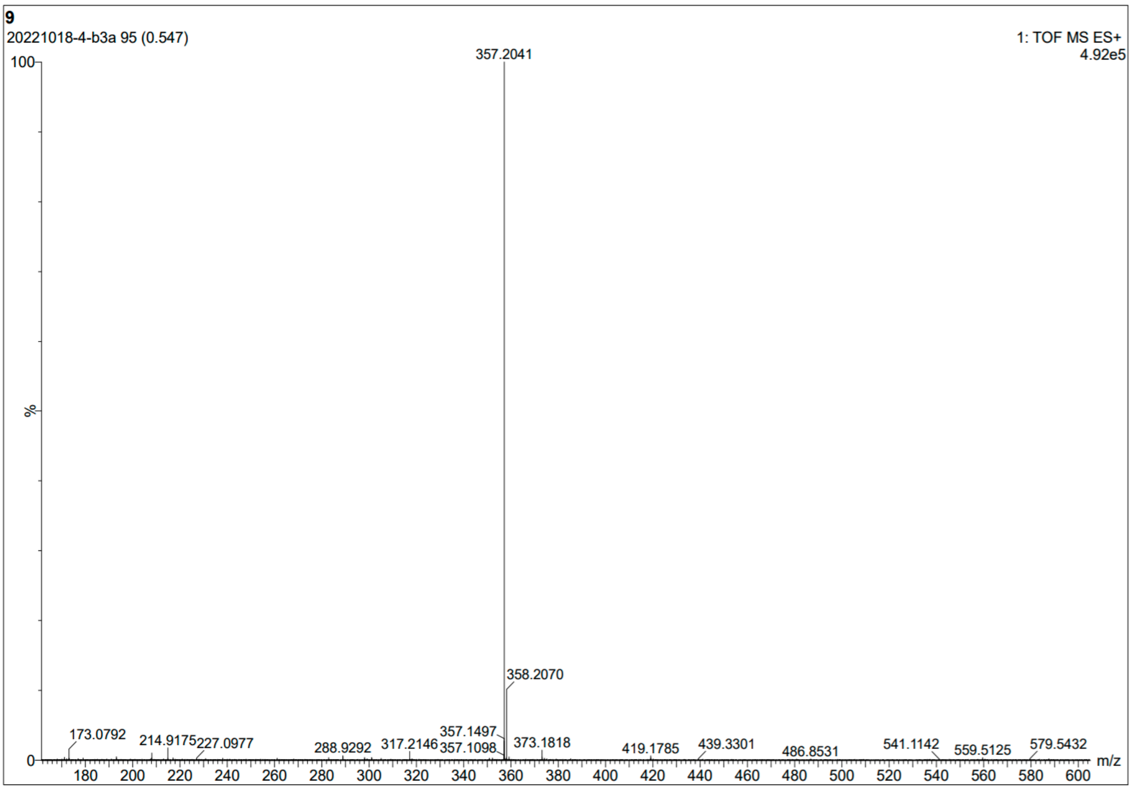

**Figure S39.** The insecticidal activity of compounds **1–5** against *T. vaporariorum* was evaluated using the leaf-dip method. All data in the graphs are the average values of three replications, corrected with control mortality using the Abbott formula. Corrected mortality (%) = (P1 − P0)/ (100 − P0) × 100, where P1 is the mortality rate in the treatment group; P0 is the mortality rate in the control group. Error bars represent standard errors of the means. Different letters indicate significant differences (ANOVA, Tukey's HSD, and $P < 0.05$).

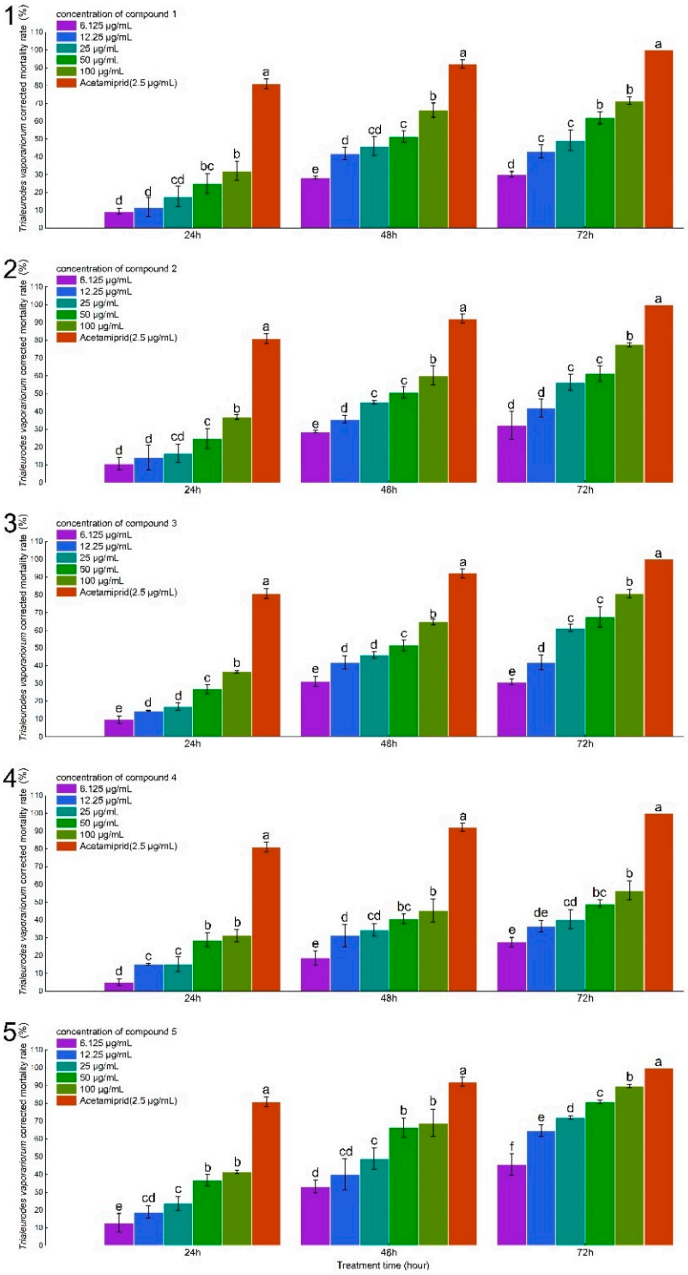

Supplement: Supplementary file 1 [file microorganisms-12-02031-s001.zip › microorganisms-3213475-supplementary.pdf]
